# Towards Real-Time and Affordable Strain-Level Metagenomics-Based Foodborne Outbreak Investigations Using Oxford Nanopore Sequencing Technologies

**DOI:** 10.3389/fmicb.2021.738284

**Published:** 2021-11-05

**Authors:** Florence E. Buytaers, Assia Saltykova, Sarah Denayer, Bavo Verhaegen, Kevin Vanneste, Nancy H. C. Roosens, Denis Piérard, Kathleen Marchal, Sigrid C. J. De Keersmaecker

**Affiliations:** ^1^Transversal Activities in Applied Genomics, Sciensano, Brussels, Belgium; ^2^Department of Plant Biotechnology and Bioinformatics, Ghent University, Ghent, Belgium; ^3^National Reference Laboratory for Shiga Toxin-Producing Escherichia coli (NRL STEC), Foodborne Pathogens, Sciensano, Brussels, Belgium; ^4^National Reference Center for Shiga Toxin-Producing Escherichia coli (NRC STEC), Department of Microbiology and Infection Control, Universitair Ziekenhuis Brussel (UZ Brussel), Vrije Universiteit Brussel (VUB), Brussels, Belgium; ^5^Department of Information Technology, IDlab, IMEC, Ghent University, Ghent, Belgium

**Keywords:** metagenomics, nanopore, Flongle, strain-level, outbreak, food surveillance, SNP analysis, STEC

## Abstract

The current routine laboratory practices to investigate food samples in case of foodborne outbreaks still rely on attempts to isolate the pathogen in order to characterize it. We present in this study a proof of concept using Shiga toxin-producing *Escherichia coli* spiked food samples for a strain-level metagenomics foodborne outbreak investigation method using the MinION and Flongle flow cells from Oxford Nanopore Technologies, and we compared this to Illumina short-read-based metagenomics. After 12 h of MinION sequencing, strain-level characterization could be achieved, linking the food containing a pathogen to the related human isolate of the affected patient, by means of a single-nucleotide polymorphism (SNP)-based phylogeny. The inferred strain harbored the same virulence genes as the spiked isolate and could be serotyped. This was achieved by applying a bioinformatics method on the long reads using reference-based classification. The same result could be obtained after 24-h sequencing on the more recent lower output Flongle flow cell, on an extract treated with eukaryotic host DNA removal. Moreover, an alternative approach based on *in silico* DNA walking allowed to obtain rapid confirmation of the presence of a putative pathogen in the food sample. The DNA fragment harboring characteristic virulence genes could be matched to the *E. coli* genus after sequencing only 1 h with the MinION, 1 h with the Flongle if using a host DNA removal extraction, or 5 h with the Flongle with a classical DNA extraction. This paves the way towards the use of metagenomics as a rapid, simple, one-step method for foodborne pathogen detection and for fast outbreak investigation that can be implemented in routine laboratories on samples prepared with the current standard practices.

## Introduction

Foodborne diseases represent a major burden worldwide (WHO, 2015). Foodborne pathogens can cause large outbreaks affecting multiple people sometimes in different regions. In case of an outbreak, the common practice of public health institutions is to investigate human cases and try to relate them to the contaminated food, in order to remove it from the food chain and prevent further contaminations. This process is called source attribution (EFSA, 2019a). This investigation consists of a microbiological and epidemiological part. In many countries, a surveillance system is also in place, screening the food chain in order to remove contaminated foodstuffs before they reach the consumer. In that case, microbial risk assessment and hazard identification are conducted, and the pathogen does not need to be linked to patient’s data, but its characteristics could be added to a database in order to conduct retrospective studies and link related cases or serve as background to detect clusters and thus putative outbreaks (ECDC and EFSA, 2019).

In both circumstances (i.e., surveillance or the microbiological part of the outbreak investigation), conventional microbiology methods based on sequential culture steps have been the standard for many years to obtain information on the bacterial contaminant(s) present in food. However, this depends on a series of steps that should be conducted on the samples, therefore requiring larger quantities of the sample that is not always easy to obtain, and most importantly, it requires obtaining an isolate, which is often time-consuming and not always successful. The heterogeneous contamination of food products, the complexity of the matrix, and the difficulty to culture certain organisms might not allow to detect a pathogen at levels as low as the infectious dose reported for human (European Food Safety Authority (EFSA), and European Centre for Disease Prevention and Control (ECDC), 2018). When an isolate is obtained, it is characterized with several (real-time) polymerase chain reactions [(q)PCRs] to detect pathogenicity markers and/or multiple locus sequencing typing (MLST), pulsed-field gel electrophoresis (PFGE), multiple locus variable-number tandem repeat analysis (MLVA), or other typing methods to relate cases of an outbreak, depending on the pathogen. This workflow does not always offer optimal resolution to discriminate the pathogenic agents at a desired level (Nouws et al., 2020a) and requires sequential tests to be conducted in the laboratory (Nouws et al., 2020a), which adds to the total cost and turnaround time of the analysis.

As an alternative, whole-genome sequencing (WGS) offers the ultimate resolution to the single-nucleotide polymorphism (SNP) level of the bacterial genome, allowing the simultaneous detection of all genes present in the bacteria as well as relatedness inference with phylogenetics (Sandora et al., 2014; Bogaerts et al., 2019), and has been recommended by the European Food Safety Authority (EFSA) for use on a list of pathogens in European laboratories (EFSA, 2014). However, circumventing the need for isolation can accelerate the collection of results even more, as well as allow the resolution of cases for which no isolate could be obtained following the detection protocol. Strain-level shotgun metagenomics approaches offer the possibility to obtain the same resolution as WGS, without the need for isolation (Forbes et al., 2017). A recent publication of the EFSA highlighted the need for demonstrating the ability of metagenomics to be used as a new alternative for risk assessment, source attribution, and outbreak investigation (EFSA, 2019b).

In our previous work, we have presented a metagenomics approach to obtain the same level of precision as the conventional bacterial detection methods and isolate’s WGS, through direct sequencing of all DNA in the sample after enrichment in a non-selective medium following the ISO standard ISO 13136:2012 (ISO: International Organization for standardization, 2012; Buytaers et al., 2020, 2021b). After short-read sequencing of 12 DNA extracts with or without removal of host DNA in a 48-h Illumina MiSeq run, we were able to link the pathogenic strains derived from metagenomics sequencing of samples containing multiple strains of the same species (*Escherichia coli*) to human isolates from the same outbreak (Buytaers et al., 2020). This was possible using a bioinformatics workflow classifying short reads to a reference genome database (Saltykova et al., 2020). Although Illumina is a widely used sequencing technology generating short reads with high accuracy, it still comes at a high cost for metagenomics, impeding a real implementation in routine. Moreover, the rather long library preparation time for multiple samples that have to be multiplexed to make the run cost-effective, as well as the 48-h sequencing run time, is not ideal for a fast response in case of an ongoing outbreak. Real-time long-read sequencing is now offered by Oxford Nanopore Technologies (ONT) with faster library preparation protocols coupled with the flexibility to cost-efficiently sequence one sample at a time on the flow cells. This could speed up the analysis of samples in an outbreak investigation and help to decrease the cost, which remains important, of metagenomics if using more cost-effective consumables for lower amounts of samples such as the MinION flow cell or the new lower output Flongle flow cell. Furthermore, long-read sequencing offers the possibility to investigate larger genome fragments without the possible bias of short-read metagenomics assembly, which could offer an added value in the context of metagenomics-based outbreak investigation.

Sequencing using Oxford Nanopore Technologies has been previously validated for the characterization of foodborne pathogenic isolates, even during the course of an outbreak (Loman et al., 2015; Quick et al., 2015; Greig et al., 2019), and has since then been tested in some metagenomics studies for pathogen identification by species and gene detection in the mixed reads (Schmidt et al., 2017; Charalampous et al., 2019). It was shown to allow attribution of potentially pathogenic taxa to the corresponding antimicrobial resistance genes they harbored by gene walkout (Leggett et al., 2020). However, strain-level characterization is necessary for the precise resolution of an outbreak, which remains a challenge for ONT metagenomics data partly due to the higher error rate of the technology (Forbes et al., 2018; Gardy and Loman, 2018). In a previous study, Hyeon et al. (2018) used an enriched food sample that was artificially contaminated with *Salmonella*, treated with immunomagnetic separation to concentrate the target bacteria, and whole-genome amplification before it was sequenced using the MinION technology. They obtained 65 and 70 SNP difference to the WGS isolate reference of the spiked bacteria after 1.5 and 48.5 h of sequencing, respectively (Hyeon et al., 2018). A similar quasimetagenomics method was used to target Shiga toxin-producing *E. coli* (STEC) and *Salmonella* in contaminated flour samples (Forghani et al., 2020). The method proved successful to cluster (without specifying the SNP differences) the metagenomics-obtained strain to the spiked isolate, for multiple single-spiked strains of each pathogen and also on samples co-spiked with one strain of each of the two pathogen species. However, this approach is still rather new, and new proofs of concept are necessary to demonstrate that it can be effectively used, possibly with a lower amount of SNP differences, for more reliable cluster definition in daily outbreak investigation. Indeed, it has not yet been tested with a non-selective enrichment method, a procedure closer to the ones currently followed by the reference laboratories (UE, 2005). Moreover, it has not yet shown its efficiency not only in samples possibly presenting multiple strains of the same species but also to cluster the metagenomics-derived strain to related human cases from the same foodborne outbreak. Finally, sequencing not only on the lower cost but also lower output, Flongle flow cell device still remains to be evaluated for such an application.

We present in this study a proof of concept of shotgun metagenomics outbreak investigation performed after ONT sequencing, combined with a new bioinformatics workflow adapted to long reads, to obtain the characterization of the foodborne pathogen at strain level in samples with various strains of the same pathogen (STEC). The spiked food samples were previously sequenced on Illumina and reported in former studies (Buytaers et al., 2020; Saltykova et al., 2020). A comparison between the results obtained with the two sequencing technologies was made. Moreover, a new approach, *in silico* DNA walking, offering the screening of food samples for pathogens at low cost based on long reads after Flongle sequencing, was evaluated after DNA extraction with or without host DNA removal. Finally, a strategy to integrate metagenomics in the current screening and pathogen characterization at the routine laboratories was proposed based on the results obtained after Flongle, MinION, and Illumina sequencing and their respective cost-effectiveness and execution time.

## Materials and Methods

### Selection of the Sample

Minced beef meat harboring a natural population of commensal *E. coli* bacteria and artificially contaminated with a low infection dose of STEC from a previous study (Buytaers et al., 2020) was used to evaluate the performance of MinION and Flongle sequencing compared to Illumina MiSeq sequencing on the same sample. Briefly, 25 g of the food matrix spiked with 5 colony-forming units (CFU) of STEC was enriched in buffered peptone water for 24 h at 37°C, following the culture described in ISO 13136:2012 for STEC detection in food (ISO: International Organization for standardization, 2012) in order to be representative of the procedures followed by the reference laboratories and therefore the samples they could get to analyze. One milliliter of the mix was used for DNA extraction using the NucleoSpin Food kit (Macherey-Nagel, Düren, Germany) or HostZERO Microbial DNA kit (Zymo Research, Irvine, CA, United States). The latter is advertised as able to remove host DNA. The strain that was chosen to artificially contaminate the food matrix was a STEC O157:H7 *eae*+, *stx1*+, *stx2*+, isolated during an outbreak in Limburg, Belgium, in 2012 (Braeye et al., 2014), and previously characterized through WGS (Nouws et al., 2020b). A negative control, a blank of the enriched food matrix, was previously sequenced on Illumina MiSeq and characterized to pinpoint the presence of commensal *E. coli* bacteria and the absence of STEC virulence genes in the meat prior to spiking (Buytaers et al., 2020; Saltykova et al., 2020).

### Oxford Nanopore MinION Sequencing

The DNA library was prepared with the Genomic DNA by Ligation protocol (SQK-LSK109; Oxford Nanopore Technologies, Oxford, United Kingdom) on the DNA extracted with the NucleoSpin kit. It was performed according to the recommendations for MinION sequencing on a MinION flow cell (R9.4.1). The prepared library was then loaded on a primed flow cell (R9.4.1), and a 48-hsequencing run was started, generating 1.2 million reads with a median length of 1,991 bp. The resulting fast5 files obtained at various sequencing time checkpoints were basecalled using Guppy version 4.2.3 (Oxford Nanopore Technologies).

### Oxford Nanopore Flongle Sequencing

Two DNA libraries were prepared, respectively, for the DNA extracted with the NucleoSpin and the HostZERO kits with the Genomic DNA by Ligation protocol (SQK-LSK109; Oxford Nanopore Technologies, Oxford, United Kingdom), following recommendations for Flongle sequencing. Each library was then loaded separately on a primed Flongle flow cell (R9.4.1), and a 24-hsequencing run was started, generating 244,019 and 187,966 reads with a median length of 686 and 3,393 bp, respectively, for the NucleoSpin and HostZERO DNA extracts. The basecalling was performed at various sequencing time checkpoints as in the “Oxford Nanopore MinION Sequencing” section.

### Long-Read Strain-Level Metagenomics Data Analysis

First, a taxonomic classification with Kraken2 (Wood et al., 2019), using the same databases (in-house database of mammals, archaea, bacteria, fungi, human, protozoa, and viruses) as used for the Illumina analysis of the same samples (Buytaers et al., 2020), was performed on the basecalled reads of MinION and Flongle sequencing, including after specific time check-points. Graphs were created on the classification results using ggplot2 in R.

Second, the presence of virulence genes in the sequenced reads and the genomic context (taxon) of the same sequencing fragment were determined using an *in silico* DNA walking method, previously described for the detection of genetically modified microorganisms using a metagenomics approach (Buytaers et al., 2021a). Briefly, a Basic Local Alignment Search Tool (BLAST) analysis was performed on all reads using BLASTn version 2.7.1 with default parameters (Camacho et al., 2009) to the databases VirulenceFinder *E. coli* (Joensen et al., 2014) and nucleotide from NCBI [Bethesda (MD): National Library of Medicine (US), 1988]. The hit to the NCBI database of each fragment presenting a virulence gene was used to obtain the genomic origin of the read harboring the virulence factors. The results were finally filtered to retain only the results for the virulence genes *stx1*, *stx2*, *eae*, and *ehxA*. For the goal of this study, focusing on a fast response to the detection of a foodborne pathogen, we presented the results obtained in the shortest timeframe necessary to obtain at least one read confirming the presence of a STEC in the sample. The results were visualized using sunburst charts.

Finally, the *E. coli* strains were inferred using Metamaps v 0.1 (Dilthey et al., 2019). Thereby, ONT reads from MinION and Flongle sequencing, including after specific time checkpoints, were classified against a database containing 2,831 reference sequences corresponding to the 976 complete *E. coli* genomes and complete 1,885 *E. coli* plasmids available from RefSeq on August 11, 2019 (O’Leary et al., 2016). Reads assigned to sub-species-level taxa were extracted.

A gene detection was conducted on the clustered reads of the inferred strains using BLAST version 2.7.1 (Camacho et al., 2009) on the VirulenceFinder *E. coli* database (Joensen et al., 2014) and SerotypeFinder O type and H type (Joensen et al., 2015) with default parameters. The strains containing *stx* genes were considered as STEC strains.

For the phylogenetic analysis, extracted reads of the STEC strain sequenced with ONT devices were mapped to a common STEC reference genome (BA000007.3) using bwa mem v 0.7.17 with the ont2d parameter set. Illumina sequences were previously analyzed through a similar workflow (Buytaers et al., 2020; Saltykova et al., 2020). Bcftools v 1.9 was used for the initial identification of potential SNPs as positions at which at least five reads contained an alternative allele, followed by filtering whereby positions with a minimal depth of 10 reads, a minimal allele frequency of 0.85, and a minimal mapping quality of 50 were retained (Supplementary Material 1). Genomic positions that did not meet the minimal sequencing depth and the minimal mapping quality criteria and potential SNPs that did not meet the minimal allele frequency were masked in the consensus sequence. Maximum likelihood substitution model selection and phylogenetic tree inference were performed using MEGA (Kumar et al., 2018), applying the nearest-neighbor-interchange (NNI) heuristic method, keeping all informative sites and using the bootstrap method with 100 replicates as a phylogeny test. The model selected was the Kimura two-parameter model with uniform rates among sites. Strains inferred from the Illumina sequencing of the same metagenomics samples [NucleoSpin extract and HostZERO extract (Buytaers et al., 2020)] and isolates from human (TIAC 1165 and TIAC 1169) and food (TIAC 1151 and TIAC 1152) originating from the same outbreak (Braeye et al., 2014), as well as some sporadic cases from the same serotype O157:H7 (TIAC 1638 and TIAC 1153), were used as background for the phylogenetic tree construction. All isolates were sequenced for a previous study (Nouws et al., 2020b). All workflows of command lines used for bioinformatics analyses in this work are presented in Supplementary Material 2.

## Results

### Long-Read Sequencing on a MinION Flow Cell for Strain-Level Metagenomics Outbreak Investigation

DNA extracted from beef meat spiked with STEC at the lowest infection dose was sequenced on a MinION flow cell. A data analysis workflow was developed in order to produce similar results as those generated with Illumina sequencing (Buytaers et al., 2020) and WGS of isolates, i.e., obtaining and characterizing the reads corresponding to the pathogenic strain in the sample and performing SNP-level phylogeny with this strain.

#### Taxonomic Classification of All Sequenced Reads

**FIGURE 1:**
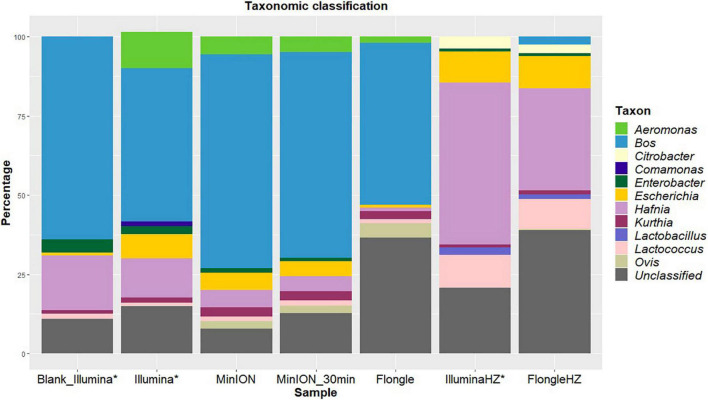
Percentages of reads classified to the genus level using Kraken2 (taxonomic classification tool) from blank and spiked beef samples extracted with two DNA extraction kits (one involving host removal, HZ) and sequenced on Illumina (MiSeq), MinION, or Flongle, with in-house databases of mammals, archaea, bacteria, fungi, human, protozoa, and viruses. The data for Illumina sequencing (*) was published in Buytaers et al. (2020). Light blue represents the proportion of “Bos” corresponding to beef reads. Yellow indicates the presence of “Escherichia” in the sample. The reads that could not be classified to the genus level for mammals, archaea, bacteria, fungi, human, protozoa, or viruses are represented in gray.

The identification of the taxa present in a metagenomics sample is an important step towards the detection of potential pathogens in a sample. In this case, a taxonomic classification was performed to the genus level on the MinION sequencing output and compared to the results previously obtained with Illumina sequencing of the same sample and of the enriched blank (unspiked) meat (Figure 1).

Beef (“*Bos*,” blue) was the main species detected in the sample after both sequencing runs (Figure 1). This was to be expected as the sample consisted of beef meat. *Ovis* (a genus that includes sheep, olive green) was classified for a small part (2%) of the reads after MinION sequencing. The bacterial genera detected were identical between the two sequencing technologies. *Escherichia*, the pathogen not only artificially spiked in the sample but also endogenously present in the beef before spiking [Blank_Illumina (Buytaers et al., 2020)], was identified for 8 and 6% of the reads after Illumina and MinION sequencing, respectively. All species were detected after 30 min of sequencing on the MinION.

#### Confirmation of the Presence of a Pathogen in the Sequenced Metagenomics Sample Using *in silico* DNA Walking on Long Reads

In order to indicate the presence of a pathogen in the sample after Illumina sequencing, a virulence gene detection was conducted on all reads (Buytaers et al., 2020). However, with that information, the virulence gene cannot be linked to the pathogen’s genome, which would be proof of the presence of the pathogen in the sample. Long-read sequencing offers the possibility to investigate the DNA fragment on which a virulence gene is detected in order to attribute it to a taxon (genomic context). This analysis is also known as *in silico* DNA walking.

As the sample was artificially spiked by a known STEC isolate, our approach was targeted at this pathogen specifically. Therefore, *in silico* DNA walking was applied to all long-read sequences with BLAST on the databases of *E. coli* virulence genes and nucleotides from NCBI, to determine if the *Escherichia*-related virulence genes, in particular *stx* genes defining an *E. coli* as a STEC pathogen, were found on *Escherichia* genome sequences, proving the presence of a pathogenic strain in the sample. This approach was tested as a fast alternative to obtain minimal characterization information on the pathogen in the sample before the inference of the strains from the metagenomics reads.

**FIGURE 2:**
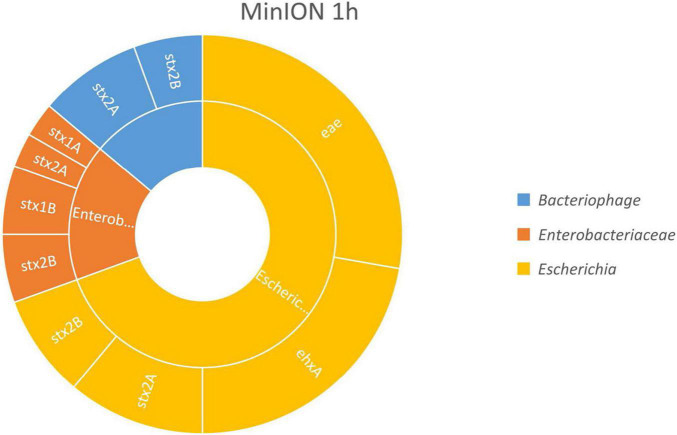
*In silico* DNA walking results, presenting the genera in the inner circle (following the color scheme specified in the legend) and the genes detected for each taxon in the outer circle for MinION sequencing of the Shiga toxin-producing *E. coli* (STEC)-spiked beef sample after 1 h of sequencing.

The results, presented in Figure 2, show that the virulence genes characteristic of the spiked STEC pathogen (*stx*, *eae*, and *ehxA*) could be linked to *Escherichia* fragments after already 1 h of MinION sequencing. This demonstrated that an *Escherichia* strain carried these genes, therefore indicating that STEC DNA was present in the samples. Moreover, as the enriched blank meat was previously sequenced and characterized (Buytaers et al., 2020), we can rule out the presence of STEC, *E. coli* virulence genes, or *stx* phages in the meat prior to the artificial contamination.

The *stx* genes (*stx1* and *stx2*) were also linked to genomic regions of *Enterobacteriaceae* and to bacteriophages. The reads assigned to *Enterobacteriaceae* could also correspond to STEC bacteria, as *Enterobacteriaceae* is the family of the *Escherichia* genus. Shorter reads may not cover any species- or genus-specific genomic features, preventing their univocal assignment to a single higher level taxon. Such reads are attributed by BLAST to a common ancestor of higher taxa from which the read could potentially be derived, e.g., the family *Enterobacteriaceae*. The same could apply to reads classified as phages, as the *stx* genes present in the STEC genome derive from the integration of these phages, but these could also be present in their mobile form in the environment.

#### Outbreak Resolution and Strain Characterization From Long-Read Sequences by Strain-Level Inference, Gene Detection, and Single-Nucleotide Polymorphism Phylogeny

**FIGURE 3:**
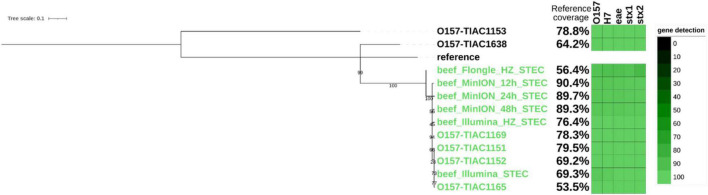
Single-nucleotide polymorphism (SNP)-based phylogenetic tree of STEC strains inferred from metagenomics sequencing (beef) and of sequenced isolates with percentage of the reference genome covered (i.e., percentage of reference genome that is useful for SNP analysis, see section “Materials and Methods”) and gene detection (O-type and H-type and genes eae, stx1, and stx2; green shaded blocks representing the query coverage) in each strain represented on the side of the branch. Isolates TIAC 1151, 1152, 1153, and 1638 are from food origin. Isolates TIAC 1165 and 1169 are from human origin. Reference: *E. coli* O157:H7 str. Sakai (BA000007.3). Green: closely related strains from the outbreak cluster. Black: sporadic cases outside the outbreak cluster. The scale bar represents nucleotide substitution per 100 nucleotide sites. Node values represent bootstrap support values.

Finally, as an equivalent to the characterization of an isolate obtained in routine, a strain-level analysis was performed on all sequenced metagenomics reads to obtain clusters of reads corresponding to the different *E. coli* strains present in the sample. The presence of the STEC strain was confirmed based on the detection of *stx* genes in the clustered reads. It corresponded to a strain mapped to the taxon 741093 from the Metamaps analysis (RefSeq NC_017906.1, NCBI:txid741093). Two other non-pathogenic strains were detected in the samples and mapped to the Metamaps proprietary taxa x494 (RefSeq NZ_CP019271.1, NCBI:txid562) and 745156 (RefSeq NZ_CP009166.1, NCBI:txid745156) (Dilthey et al., 2019). Metamaps uses an extended database taxonomy where some NCBI taxonomic nodes are further subdivided to ensure higher resolution of taxonomic assignment. The same strains were detected after Illumina sequencing (Saltykova et al., 2020). The STEC strain was further investigated for SNP phylogeny to relate it to other cases (i.e., isolates from food and human origin related to the same outbreak as the spiked isolate and sporadic cases). Strains inferred from Illumina sequencing of the same sample (Buytaers et al., 2020) were also included in the tree (Figure 3). The inferred STEC strain obtained after 12, 24, and 48 h of MinION sequencing clustered with the corresponding isolates and metagenomics strain obtained from Illumina sequencing, with 0 SNPs distance (Supplementary Material 3), and separated from the sporadic cases. The presence of three virulence genes of importance for STEC characterization (*eae*, *stx1*, and *stx2*), as well as the serotyping genes (O-type and H-type), was also confirmed in the genome of the inferred STEC strain. The serotype and virulence genes in the inferred STEC strain correspond to the genes present in the strain that was spiked. The reference coverage from the MinION run starting from 12 h of sequencing was comparable to the coverage obtained from isolates of the same outbreak and strain inferred from Illumina metagenomic sequencing and therefore considered as sufficient for a phylogenetic analysis. Shorter sequencing time on the MinION did not offer sufficient coverage to conduct the phylogenetic analysis (Supplementary Material 3).

### Investigation of Long-Read Flongle Sequencing as a Less Expensive Alternative for Strain-Level Metagenomics Outbreak Investigation

The same sample of beef meat containing an endogenous population of non-pathogenic *E. coli* and spiked with a STEC pathogen, previously characterized to the strain level after Illumina sequencing (Buytaers et al., 2020), was sequenced on a Flongle flow cell to investigate a less-expensive alternative. However, as the output of the Flongle is approximatively 10 times lower than the MinION, we also sequenced on the Flongle DNA for which the extraction involved host removal, previously sequenced on Illumina (Buytaers et al., 2020), in an attempt to increase the amount of reads linked to the microbial pathogen. The data analysis on the sequenced long reads was the same as the data analysis presented for the long reads sequenced on the MinION. The analysis was also conducted at different time points of the Flongle sequencing run to determine the time needed to achieve the expected results.

#### Taxonomic Classification of All Sequenced Reads

After Flongle sequencing, the main genus detected in the sample without host DNA removal was *Bos* (Figure 1). The same bacterial taxa, with the exception of *Enterobacter*, were detected as for the Illumina and MinION sequencing, including *Escherichia*, but with a higher percentage of unclassified reads. As for MinION sequencing, a small portion (5%) of mammal reads were incorrectly classified as *Ovis*.

The DNA extract treated with host DNA removal agent (FlongleHZ) presented 2% of reads classified as *Bos* and 0.5% of reads classified as *Ovis*, although no reads were classified as mammals in the Illumina sequencing of the same DNA extract. However, this is a large decrease compared to the amount of Flongle reads classified as eukaryotes without the host DNA removal step (50%). The bacterial taxa detected were the same for this sample after Illumina or Flongle sequencing and, except for the absence of *Aeromonas* and *Comamonas* and the presence of *Citrobacter* and *Lactobacillus*, were identical to the bacterial taxa detected without host DNA removal. This difference might be explained by the presence of bacterial DNA in the extraction buffer or its presence at very low level in the food sample. *Escherichia* represented 10% of the reads, which is slightly higher than the values obtained without host DNA removal. The sample with host DNA removal sequenced on the Flongle presented the highest percentage of unclassified reads (39%).

#### Confirmation of the Presence of a Pathogen in the Flongle-Sequenced Metagenomics Sample Using *in silico* DNA Walking on Long Reads

Similar as for MinION sequencing, an *in silico* DNA walking was conducted in order to attribute a genomic context (taxon) to detected virulence genes. This analysis was conducted on all reads generated at different time points during the Flongle sequencing of the two DNA extracts (with or without host DNA removal).

**FIGURE 4:**
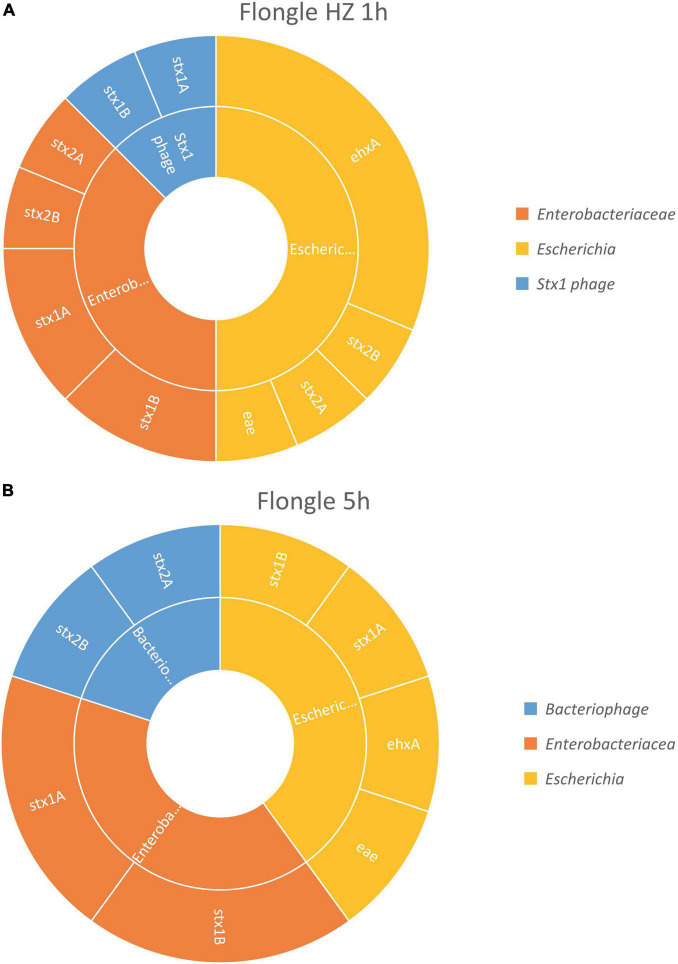
*In silico* DNA walking results, presenting the genera in the inner circle (following the color scheme specified in the legend) and the genes detected for each taxon in the outer circle of the STEC-spiked beef sample after DNA extraction with or without host removal. **(A)** Flongle sequencing after 1 h of sequencing, DNA extract with host removal. **(B)** Flongle sequencing after 5 h of sequencing, DNA extract without host removal.

After 1 h of sequencing, the virulence genes characteristic of a STEC (i.e., *stx*, *eae*, and *ehxA*) could be retrieved in the sample treated with host DNA removal (Figure 4A) and detected on genome fragments that could be assigned to *Escherichia*. Similarly as with the MinION analysis, the virulence genes were also found associated in smaller proportions to *Enterobacteriaceae*, which correspond to the family of the *Escherichia* genus, or *stx1* phage, the bacteriophage carrying the *stx1* gene that can be inserted in the STEC genome. The classification to a higher level (*Enterobacteriaceae* or phage) might be explained by the short length of the reads.

Without host DNA removal (Figure 4B), 5 h of sequencing were sufficient to obtain the required information to determine that the pathogen was present in the sample, i.e., virulence genes *stx*, *eae*, and *ehxA* associated to *Escherichia* genome. Again, the virulence genes could be also assigned to *Enterobacteriaceae*, as well as bacteriophage for some *stx2* genes.

As a STEC is defined as an *E. coli* harboring an *stx* gene, the information presented was sufficient to conclude that a STEC was present in the samples, after 1 h of sequencing with host DNA removal and 5 h of sequencing without host DNA removal. However, a longer sequencing time would be required to obtain all virulence genes characterizing the strain that was spiked. In our workflow, the full characterization of the STEC present in the sample is done at the next step, after strain inference, in order to characterize specifically each potential pathogenic strain present in the sample.

#### Outbreak Resolution and Strain Characterization From Flongle Long-Read Sequences by Strain-Level Inference, Gene Detection, and Single-Nucleotide Polymorphism Phylogeny

The different *E. coli* strains present in the sample were inferred from the reads of the two Flongle sequencing runs, and the STEC strain was identified among these strains after detection of *stx* genes in the clustered reads (Supplementary Materials 4, 5).

The pathogenic strain corresponded to reads that mapped to the Metamaps taxon 741093 (RefSeq NC_017906.1, NCBI:txid741093) for the DNA extract without host DNA removal, i.e., a similar strain as found with MinION sequencing, and to Metamaps taxon x13 (RefSeq NZ_CP012802.1, NCBI:txid83334) for the DNA extract with host DNA removal, which is also a STEC O157:H7. The endogenous strains were mainly mapped as Metamaps taxon 745156 (RefSeq NZ_CP009166.1, NCBI:txid745156), similarly as for the MinION sequencing, as well as Metamaps taxon x311 (RefSeq NZ_CP019267.1, NCBI:txid562) for the extract without host DNA removal (Supplementary Materials 4, 5).

After SNP calling, it was observed that the coverage of the reference genome (Supplementary Material 3) was insufficient to conduct a SNP-level phylogenetic analysis (less than 1%) for the DNA extract without host DNA removal. Therefore, the inferred STEC strain obtained after Flongle sequencing of the DNA extract without host DNA removal was not included in the phylogenetic tree. However, 24 h of Flongle sequencing of the DNA extract with host DNA removal led to obtaining clustered reads covering 56% of the genome at or above 10× coverage, which was sufficient to cluster the metagenomics-derived strain with the outbreak cases on the phylogenetic tree (Figure 3). Serotyping genes (O-type and H-type) as well as virulence genes *eae*, *stx1*, and *stx2* could be detected with high identity in the strain, confirming that it was similar to the spiked strain. A distance of 0–3 SNPs per million genomic positions (Supplementary Material 3) was observed for the other isolates from the same outbreak as well as the metagenomics-derived strains from Illumina or MinION sequencing, which is in the expected range. However, the distances of the outbreak strain to the background isolates (TIAC 1153 and TIAC 1638) were somewhat lower with Flongle sequencing data after host DNA removal than with MinION and Illumina sequencing data (30 SNPs per million of genomic positions for the Flongle sequencing compared to 39–46 SNPs to TIAC 1638 for Illumina and MinION, and 126 SNPs per million of genomic positions for the Flongle sequencing compared to 139–155 SNPs to TIAC 1153 for Illumina and MinION; Supplementary Material 3), indicating that not all SNPs could be called at the obtained coverage.

## Discussion

The rapid and precise characterization of a pathogen during foodborne outbreak investigation, as well as the tracing back to the food source, is crucial to stop further spreading of the infections. Therefore, a metagenomics approach has been proposed as an alternative to the currently performed microbiological analyses requiring a not-always-straightforward isolation of the pathogenic strain. As previously described (Buytaers et al., 2020), Illumina sequencing may be used to obtain the full information necessary for outbreak investigation from metagenomics samples, without the need for isolation, and this to the strain level, after about one full week of lab work (Buytaers et al., 2021b). However, not only the need for more proofs of concept but also the high cost of such an analysis impact its potential implementation as a routine practice. To render the analysis somehow more cost-effective, while still taking the required coverage into account, 8–12 samples were pooled into one Illumina MiSeq run in previous studies (Leonard et al., 2016; Buytaers et al., 2020, 2021b). However, it might not always be possible to analyze this number of samples as the number of available food samples during outbreak investigation varies and is not gathered at a single time point. Besides, delaying the sequencing run to gather sufficient samples is not an option when a fast response is required, especially in outbreak investigation. Using a smaller number of samples in the run would however substantially increase the sequencing cost per sample. Moreover, these runs, generating 2×250-bp reads, have a set sequencing duration of 48 h, which is significant during ongoing outbreak investigations. Long-read sequencing and flexibility in sequencing time, which is made possible by ONT, could offer a solution to these drawbacks.

In this study, we first sequenced an artificially contaminated sample (beef containing an endogenous community of non-pathogenic bacteria including *E. coli*, spiked at very low dose with STEC), previously sequenced on Illumina (Buytaers et al., 2020), with a MinION flow cell. The data analysis followed the same flow as the analysis previously described for Illumina sequencing (Buytaers et al., 2020), but with adapted algorithms and tools for taxonomic classification, virulence gene detection, and genome inference of long reads. This allowed to match the contaminated food with human isolates from the same outbreak, after a shorter sequencing time. After only 12 h of sequencing, endogenous and pathogenic *E. coli* strains could be obtained from the sequenced reads, and the clustered reads corresponding to the STEC could be linked to outbreak isolates from food and human origin. The virulent strain-related reads harbored all virulence genes expected from the spiked bacteria and could be placed accurately in a phylogenetic tree with 0 SNP difference to the outbreak cluster, which is much lower than the SNP distance previously obtained after metagenomics ONT sequencing (Hyeon et al., 2018). The high number of SNPs observed by Hyeon et al. (2018) could be due to the specificity of the SNP calling procedure that was used. The authors applied a workflow based on the CFSAN pipeline, with a relatively low minimal allele frequency for SNP calling (0.6). These settings have, however, shown to exhibit a lower SNP calling accuracy even with the more accurate Illumina sequencing data, with higher SNP distances between the outbreak isolates as a result (Saltykova et al., 2018). Moreover, the long reads sequenced with ONT also offer the possibility to investigate at the same time the genomic context of reads carrying these virulence genes, in an *in silico* DNA walking approach. Recently, the European authorities proposed the attribution of the virulence genes to their respective bacterial host as an isolate-free alternative to confirm the presence of foodborne pathogens after metagenomics sequencing (EFSA, 2019b). We were able to detect the expected virulence genes (*stx*, *eae*, and *ehxA*) on genome fragments that could be linked to an *Escherichia* genome, therefore confirming the presence of the pathogen in the sample after only 1 h of sequencing on the MinION flow cell. This approach could eventually be implemented in real time while receiving data from the sequencer, as it has been shown previously for AMR genes (Leggett et al., 2020).

MinION sequencing offers the opportunity to work with long reads, allowing access to the genomic context of the reads sequenced, as well as the flexibility of real-time sequencing and sequencing one sample at a time. However, it remains an expensive consumable, and therefore, the lower cost Flongle flow cell was also tested. The Flongle flow cell was ideal to rapidly obtain a confirmation of the presence of a pathogen in the sample at the lowest cost after taxonomic classification and *in silico* DNA walking. Indeed, it allowed to confirm the presence of the STEC strain (detection of *stx* gene in *Escherichia* genome) after 1 h of sequencing if host DNA removal was conducted or 5 h with traditional DNA extraction. As the output of the Flongle flow cell is substantially lower compared to the output of the MinION flow cell, retrieval of information for strain comparison was only possible when host DNA removal was conducted during the DNA extraction. The coverage of the reference genome by the clustered reads corresponding to the STEC strain obtained from the extract without host DNA removal was not sufficient to establish phylogenetic links. The threshold to determine if a strain contains sufficient reads using metagenomics to perform further characterization or SNP phylogeny is hard to define strictly as lower coverages are also observed for genomics on isolated strains (e.g., TIAC 1165 covering 54% of the reference genome, Figure 3). More analyses such as the one within this study, including for other pathogens, are necessary to pinpoint such limits. We could also observe that the reference genome to which the reads were assigned after Flongle sequencing was different from the reference genomes mostly covered after MinION or Illumina analyses. However, the different references to which the STEC reads clustered were all STEC O157, and the interpretation of the results was not impacted by the reference (SNP-level phylogeny obtained for the different strains). A future alternative could be to pool reads assigned to groups of similar references instead of working with individual references, as already proposed in the work of Saltykova et al. (2020) for short-read sequences.

**FIGURE 5:**
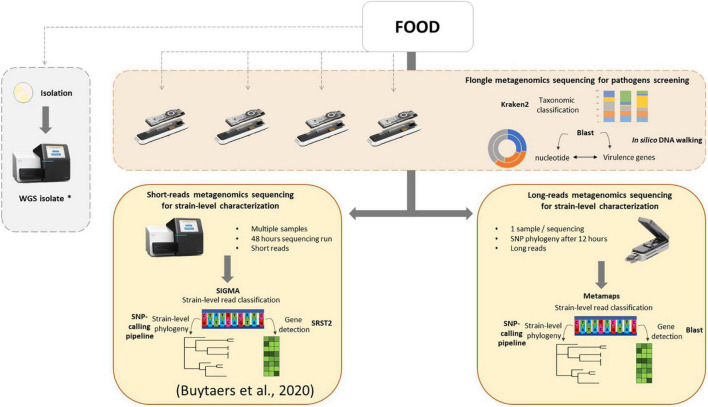
Integrated metagenomics-based strategy for microbiological foodborne outbreak investigation. As first optional steps, food samples can be screened for the presence of bacterial pathogens using metagenomics Flongle sequencing, taxonomic classification, and *in silico* DNA walking (based on BLAST to the nucleotide database and virulence genes database) in parallel with the ongoing attempt to isolate the pathogen, followed by WGS of the obtained isolates. A strain-level characterization can be attempted from the Flongle sequencing or conducted after using an Illumina strategy (more cost-effective for multiple samples) or a MinION strategy (fast response for one sample) in food samples for which the presence of a pathogen is confirmed. The strain-level data analysis for Illumina sequencing was previously presented Buytaers et al. (2020). The strain-level data analysis workflow for MinION sequencing is based on classification using Metamaps, a gene detection with BLAST, and phylogenetics with a SNP-calling pipeline. The asterisk (*) indicates that WGS isolate data is interesting to feed to reference genome databases for the classification of the metagenomics reads for future analyses.

In the present work, a three-step analysis has been applied on food samples sequenced with different flow cells. For each step, the minimal time required to obtain results was assessed. First, a taxonomic classification to obtain an overview of the genetic content of the food sample, followed by virulence gene detection coupled to an *in silico* DNA walking method for hazard identification. These tests could be performed in a very fast timeframe of a few hours, depending on the treatment of the DNA extract and the selected sequencing flow cell, and could even be implemented in real time in the future. This could potentially solve partially, i.e., when food leftovers are available and it concerns a bacterial origin (for which isolation is currently the routine approach), the issue of foodborne outbreaks for which the food source cannot be determined, accounting for 60% of all (i.e., also including other agents as source) outbreaks notified in the EU (EFSA, 2021). Finally, as a third step, strain-level phylogeny in order to relate human cases of an outbreak to its food source can be achieved after 12 h of sequencing on a MinION flow cell or 24 h on a Flongle flow cell if host DNA removal was applied during the DNA extraction. Notably, for *in silico* DNA walking, a threshold of one read harboring an *stx* gene and traced back to the *Escherichia* genus was considered as sufficient to determine the minimal time to suspect the presence of a pathogen in the sample. However, a discussion within the international scientific community is necessary to determine such threshold, and we recommend to continue the sequencing after this minimal time to collect more information. Moreover, obtaining and characterizing the pathogenic strain (third step) are still necessary to confirm the suspicion. Based on this work, a new strategy for detection of bacterial pathogens in food, using shotgun metagenomics, could be proposed to the reference laboratories (Figure 5): a screening of all food samples that might be related to a foodborne outbreak, including those for which the contaminant is unknown, for pathogens using the Flongle and taxonomic classification followed by virulence gene detection and *in silico* DNA walking, potentially in real time. This might involve additional enrichment media and/or conditions to be able to cover all bacterial foodborne pathogens, depending on the specific outbreak based on patient’s symptoms, to fully replace the conventional way of working. Once the presence of a bacterial pathogen is confirmed in a food sample, this analysis can be followed by a strain-level read classification and phylogeny that can be attempted on the Flongle sequencing data or, if not possible, based on further Illumina or MinION sequencing. Also, the choice of the sequencing technology will depend on a cost analysis based on the amount of samples to sequence as well as the timeframe to obtain results and the capacities of the laboratory. This strategy could be run in parallel with attempting to obtain a bacterial isolate from the same food samples, which would ideally be sequenced with WGS in order to populate the still necessary databases that are required to perform the metagenomics-based bioinformatics analyses. The perspective of such a strategy consolidates the new perception that metagenomics has the ability to be used as a new alternative for outbreak investigation, source attribution, and risk assessment of foodborne microorganisms (EFSA, 2019b). In order to implement the same data analysis applied to artificially STEC-contaminated samples in this study to other bacterial pathogens, the same workflow can be followed, and only the databases for gene detection and read mapping have to be adapted according to the contaminant(s) detected through taxonomic classification.

The implementation of such a metagenomics approach in routine, however, still requires overcoming several challenges. First, the data analysis currently requires sufficient informatics hardware, especially performant GPUs for real-time base-calling and analysis. Additionally, trained bioinformaticians are needed, as no automated pipeline has been developed yet for a strain-level pathogen characterization. Benchmarking studies comparing more bioinformatics tools need to be performed to identify tools allowing to obtain similar results in the same, or even faster, timeframe. For this, we believe that studies such as this one offer interesting datasets to be explored further. Second, some consumables like the Flongle flow cells, which have a very short storage life, can be difficult to obtain in a short timeframe when the demand exceeds the production capacities as experienced during the COVID-19 pandemic. The output of Flongle flow cells might also be difficult to predict due to the possible instability of the very low amount of pores not only before loading but also after loading. This might affect low-level contamination samples. The MinION sequencing resulted in a larger output, allowing the potential applicability to other samples, regardless of the quality of the flow cell (number of pores). The work of Forghani et al. (2020) showed that a quasimetagenomics method to the strain level with MinION sequencing can be extended to other strains of STEC and other bacterial pathogens without a problem. However, our study, although only including one serotype, showed the potential of the metagenomics approach for samples presenting a population of several different *E. coli* strains (including non-pathogenic strains). More studies might be necessary to validate the potential of long-read strain-level metagenomics for food safety assessment and foodborne outbreak investigation for other pathogens, including viruses and parasites. The enrichment and extraction methods might have to be adapted depending on the pathogens to investigate. Moreover, while we analyzed samples contaminated with the lowest infectious dose, more studies with different contamination loads might lead to a more precise limit of detection for the method, especially as the number of pathogenic cells is undetermined after enrichment.

In conclusion, this work is a proof of concept of the potential to conduct real-time and affordable strain-level outbreak investigation based on ONT long reads, testing the potential of the MinION as well as the Flongle flow cells. Although a limited amount of samples and only one STEC strain was included in our proof-of-concept study, we demonstrated the ability to obtain the characterization and relatedness of a STEC spiked at a very low dose in a food matrix based on metagenomics sequencing on a MinION flow cell after only 12 h or on a Flongle flow cell after 24 h if host DNA removal was applied during the DNA extraction. Moreover, we also presented a rapid strategy to confirm the presence of a pathogen in a food matrix based on long-read sequencing without the need for isolation (i.e., *in silico* DNA walking). All this was possible on food samples enriched in a non-selective medium following the ISO practice. This makes it particularly interesting for reference laboratories when only limited quantities of the food samples are left, and there is no need for sequential culturing steps on pathogen-specific selective media, with pathogen-specific growth conditions. Moreover, with the method we propose, food that has been enriched at the reference laboratory can be sequenced with a metagenomics workflow in parallel to the isolation protocol, without the need for a different enrichment protocol, which can be particularly interesting when no isolate can be obtained. Finally, these results allowed proposing a more global perspective, as a metagenomics-based strategy to be used by the routine (reference) laboratories, determined by the required level of information required, cost-effectiveness, and timeframe to obtain results. This contributes to the demand of the EFSA asking to demonstrate the ability of metagenomics to be used as a new alternative for risk assessment, source attribution, and outbreak investigation (EFSA, 2019b).

## Data Availability Statement

All sequencing data is publicly available at NCBI SRA under BioProject PRJNA736700.

## Author Contributions

FB, AS, and SDK conceptualized the study, conducted the formal analysis, conducted the investigation, were responsible for the methodology, and wrote the original draft. FB, SD, BV, and DP curated the data. NR and SDK were involved in the funding acquisition and were involved in project administration. FB, SD, BV, KV, NR, and DP provided the resources. FB, AS, and KV were responsible for the software. KM and SDK supervised the study. SD, NR, DP, and SDK performed the validation. FB was responsible for the visualization. All authors reviewed and edited the manuscript, contributed to the article, and approved the submitted version.

## Conflict of Interest

The authors declare that the research was conducted in the absence of any commercial or financial relationships that could be construed as a potential conflict of interest.

## Publisher’s Note

All claims expressed in this article are solely those of the authors and do not necessarily represent those of their affiliated organizations, or those of the publisher, the editors and the reviewers. Any product that may be evaluated in this article, or claim that may be made by its manufacturer, is not guaranteed or endorsed by the publisher.

## Supplementary Material

Supplementary Material 1 Reference coverage and allele distribution in the inferred strains from long-reads metagenomics sequencing.

Supplementary Material 2 Workflow of command lines for bioinformatics analyses used in this study.

Supplementary Material 3 Reference coverage and total and relative SNP distances (per million genomic positions) of the STEC phylogenetic tree.

Supplementary Material 4 Metamaps result (STEC reference genome assignment of the reads) after Flongle sequencing.

Supplementary Material 5 Metamaps result (STEC reference genome assignment of the reads) after Flongle sequencing and host DNA removal.
