# Supplementary material for: Towards Real-Time and Affordable Strain-Level Metagenomics-Based Foodborne Outbreak Investigations Using Oxford Nanopore Sequencing Technologies
Source: Front Microbiol. 2021 Nov 5;12:738284. doi: 10.3389/fmicb.2021.738284 (PMC8602914; doi:10.3389/fmicb.2021.738284)
Supplement: Supplementary Material 1 — Reference coverage and allele distribution in the inferred strains from long-reads metagenomics sequencing. [file Table_1.DOCX]

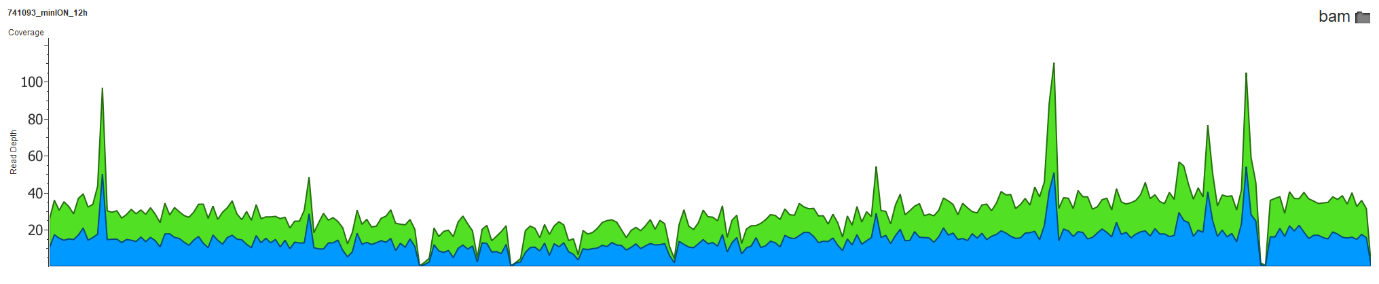


**Minion 24h 741093: Average = 40.0845x**

**Minion 12h 741093: Average = 29.9528x**


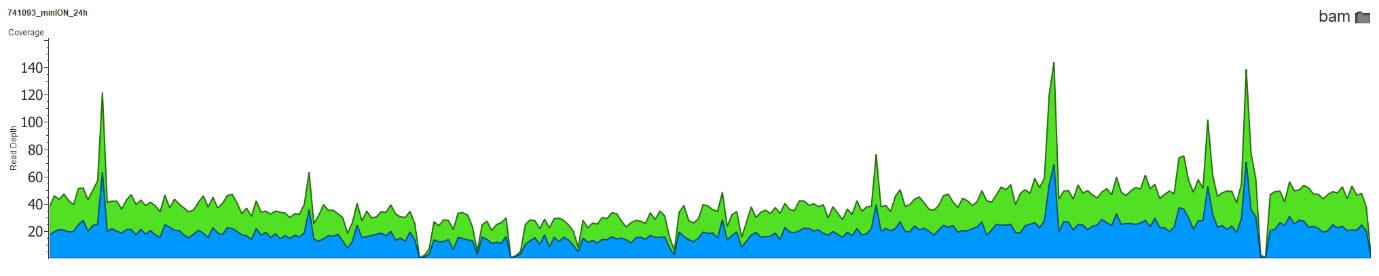


**Minion 48h 741093: Average = 43.0755x**


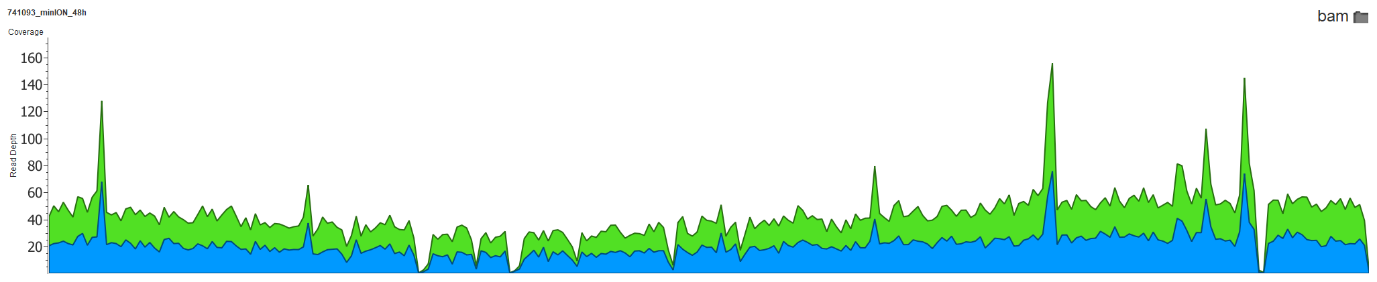


**FlongleHZ 24h x13: Average = 10.6075x**


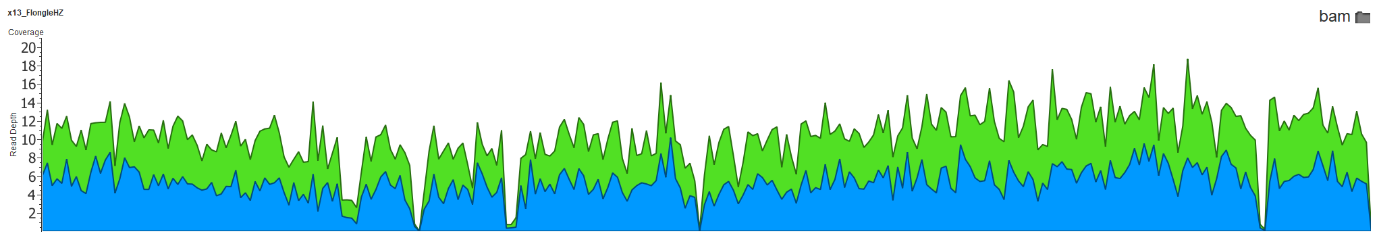


Figure S1: Coverage of the the long-read samples corresponding to the spiked STEC strain determined using BA000007.3 STEC reference genome. Green and blue colours represent forward and reverse reads, respectively.


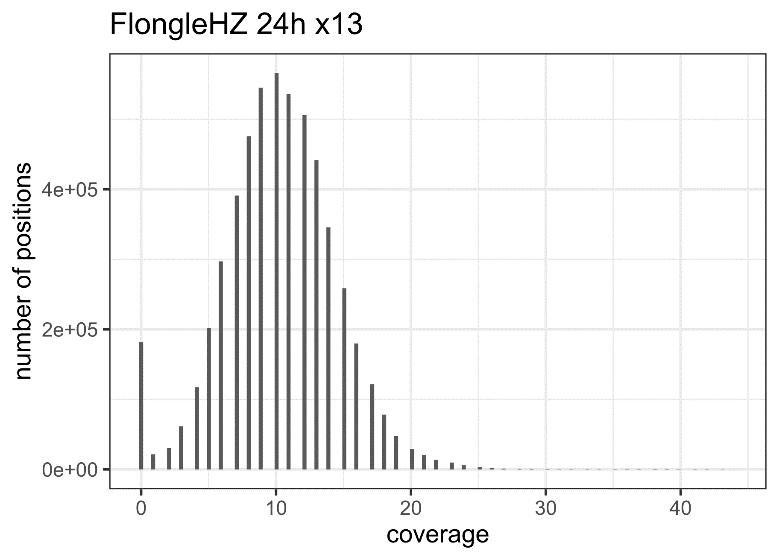

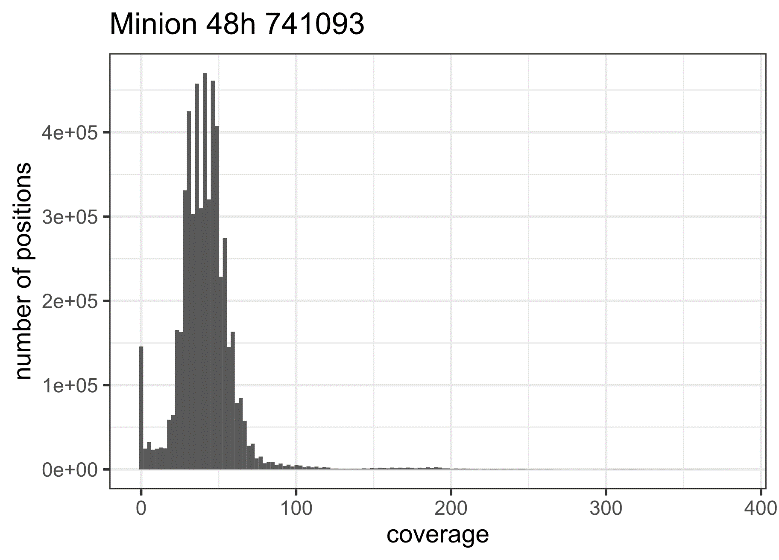

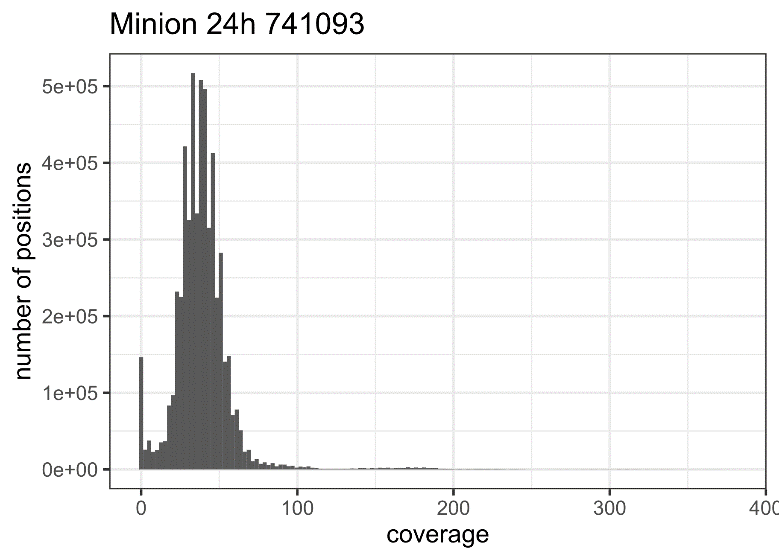

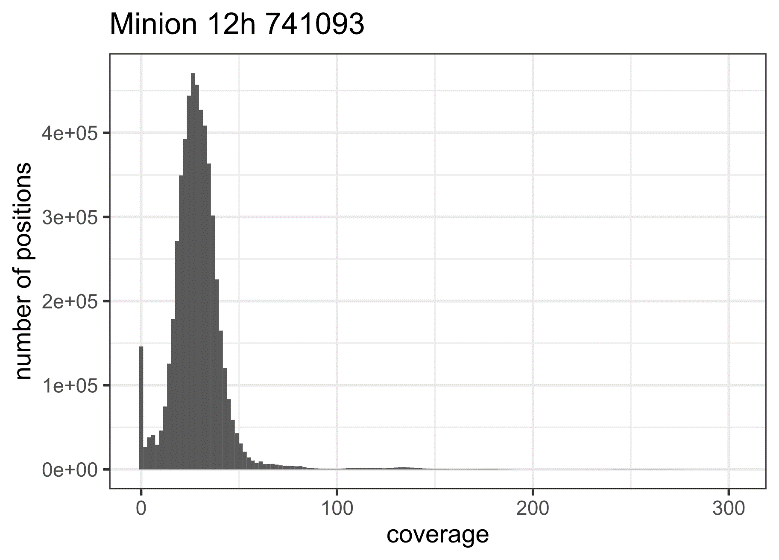


Figure S2: Coverage distribution histograms of the the long-read samples corresponding to the spiked STEC strain determined using BA000007.3 STEC reference genome.

Figure S3 (below): Alternative allele distribution at the potential SNP positions of different coverage in the long-read samples corresponding to the spiked STEC strain. Potential SNP positions include genomic positions with at least 5 reads containing the same alternative allele call. Regions with a mapping quality lower than 50 are omitted. A: 2D histogram of the frequency of the alternative allele and coverage at potential SNP positions. Number of positions are represented by colour and size of the dots. Alternative allele frequency (0.85) and coverage (10x) thresholds used for filtering high-quality SNPs are indicated with dotted lines. B: Alternative allele frequency histogram at all potential SNP positions. C: Zoom in at the region of B containing potential high-quality SNPs. D: Alternative allele frequecy histogram at potential SNP positions meeting the coverage threshold for determination of high-quality SNPs (at least 10 reads). E: Zoom at the region of D containing potential high-quality SNPs.


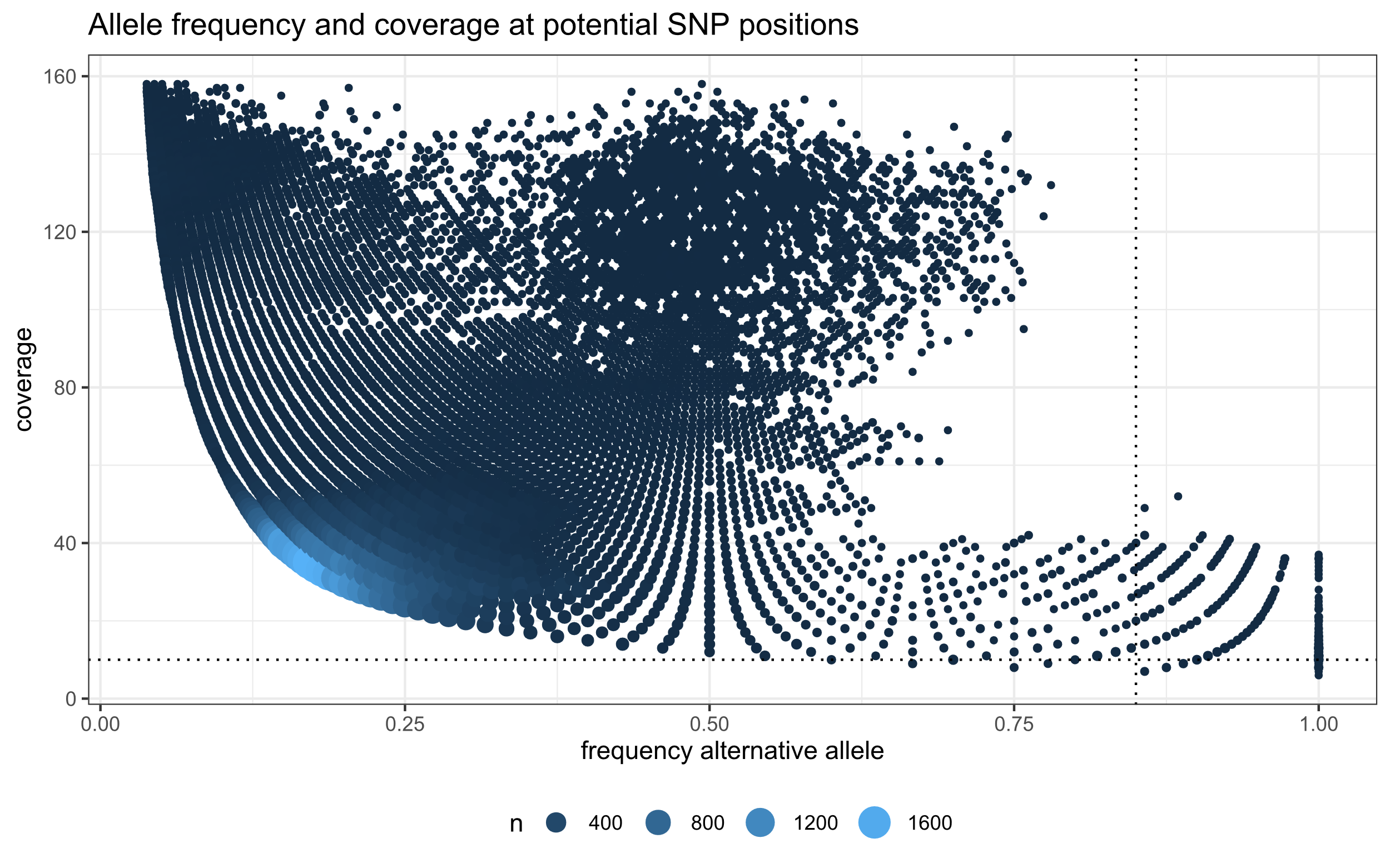


**Minion 12h 741093**

**A**


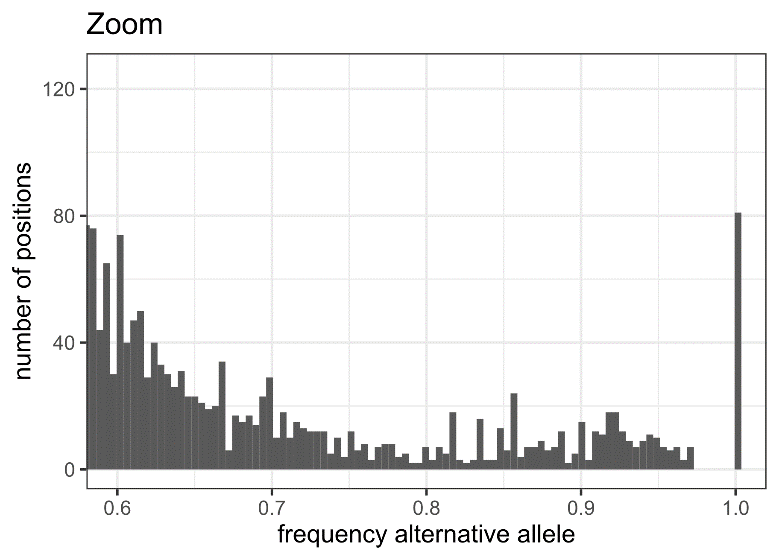

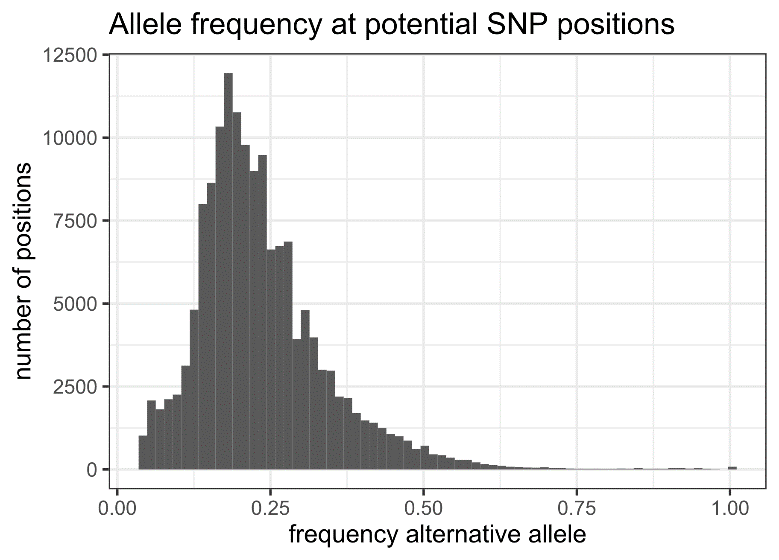


**C**

**B**


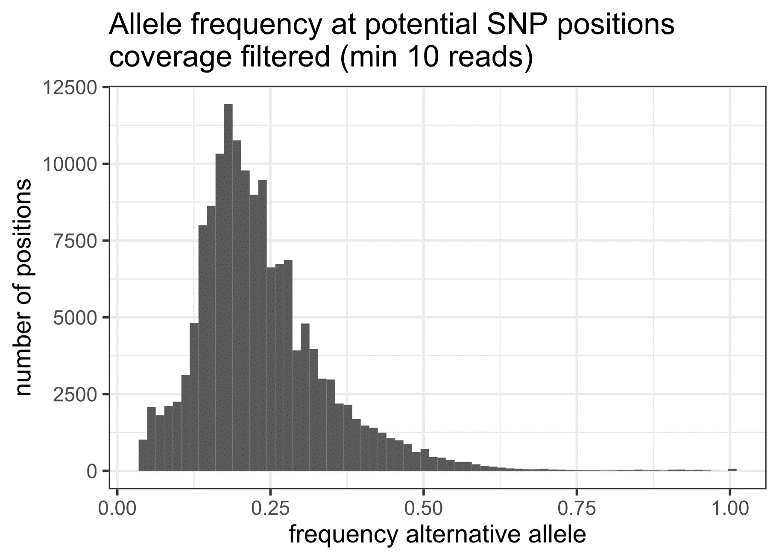

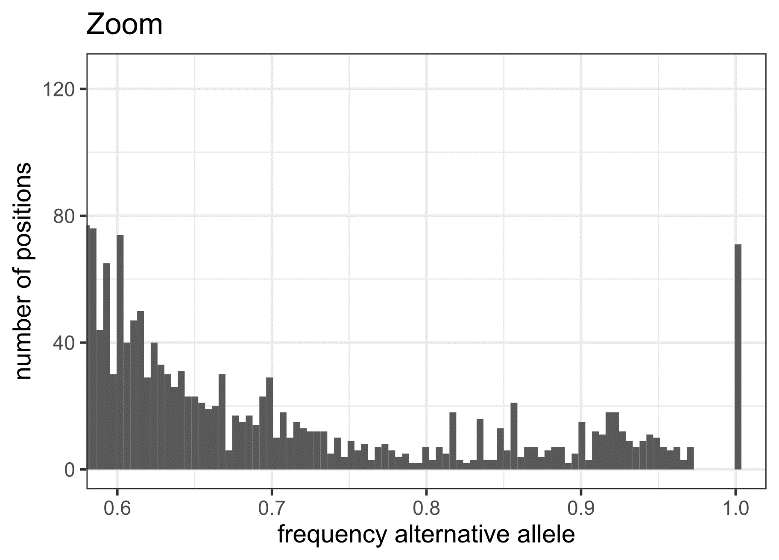


**E**

**D**


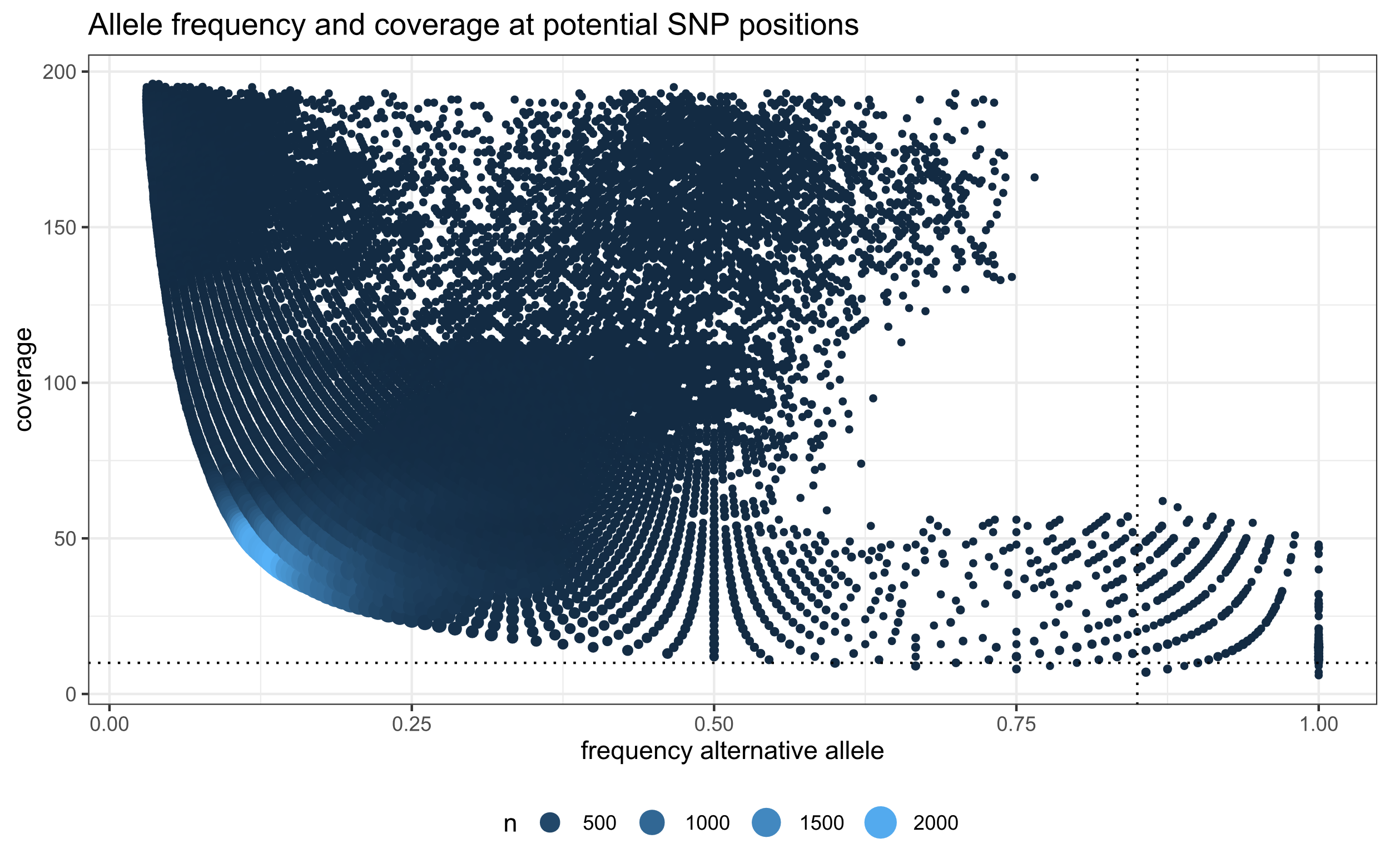


**Minion 24h 741093**

**A**


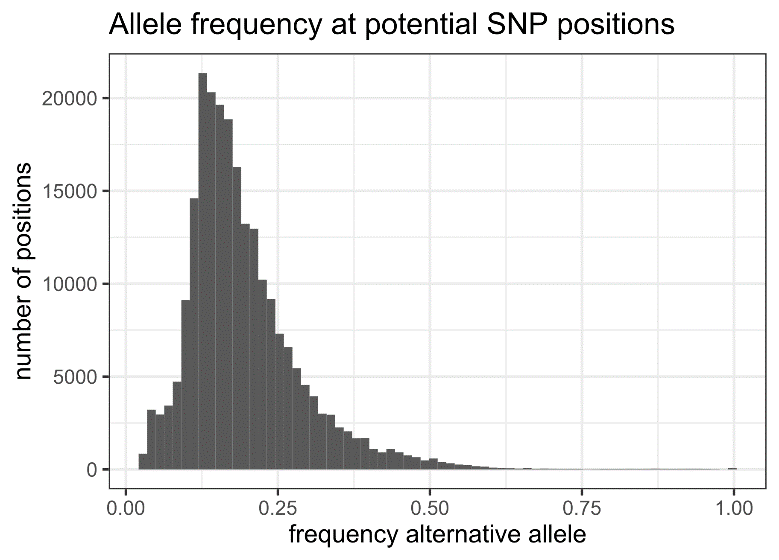


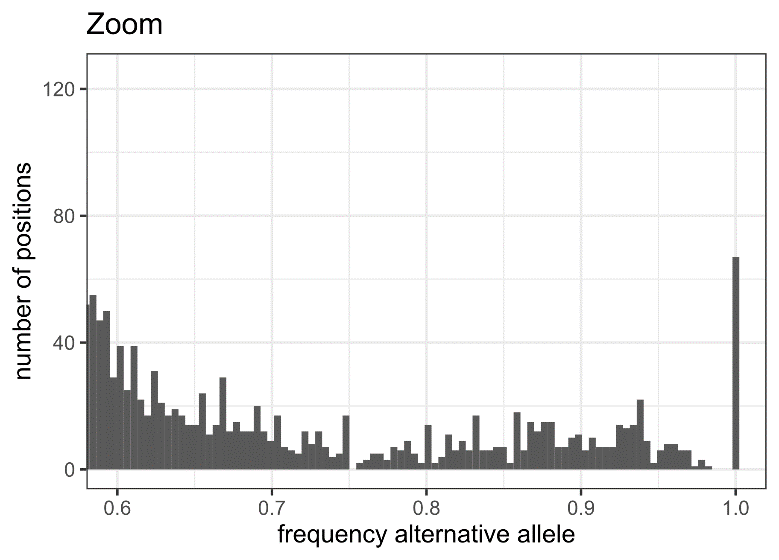


**C**

**B**


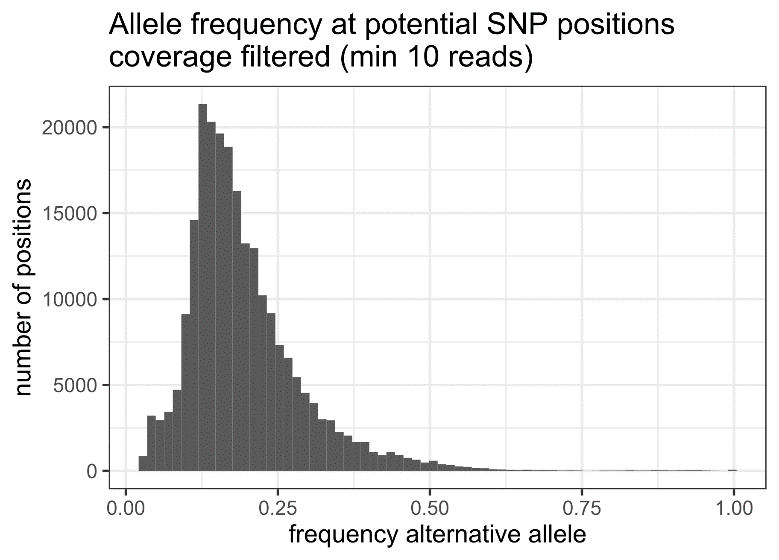

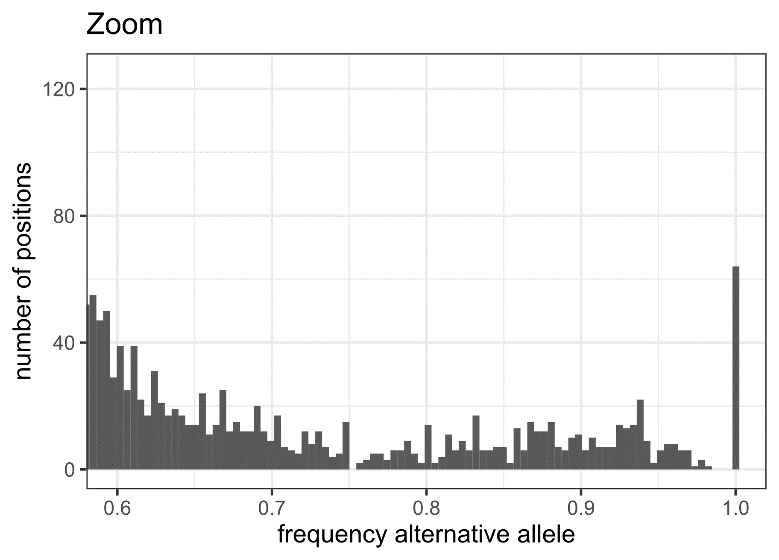


**E**

**D**


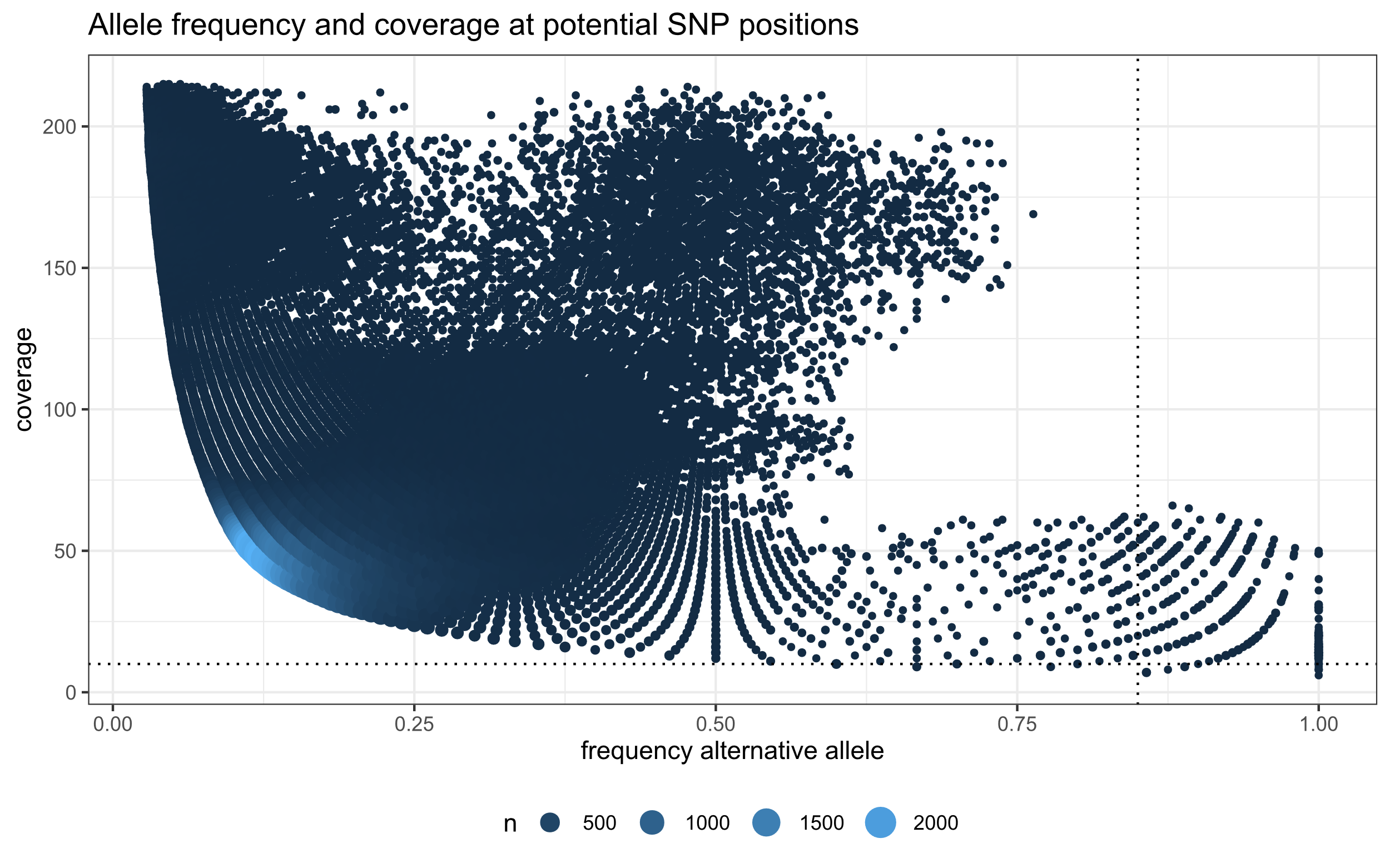


**Minion 48h 741093**

**A**


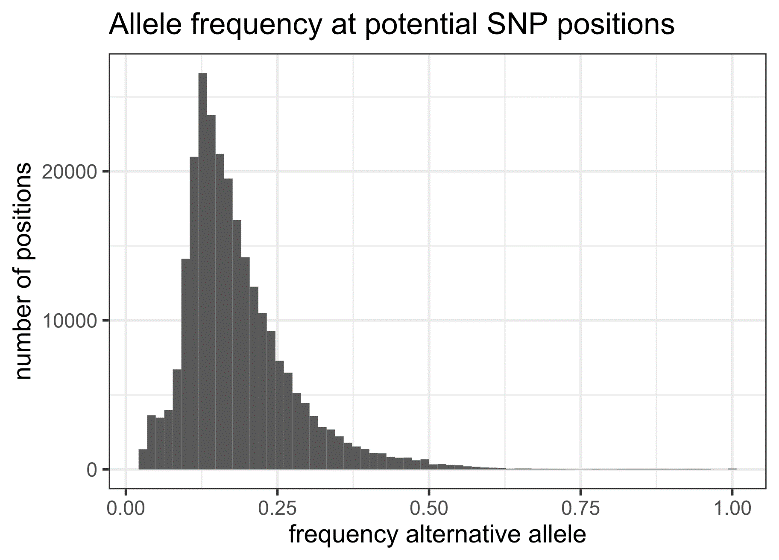

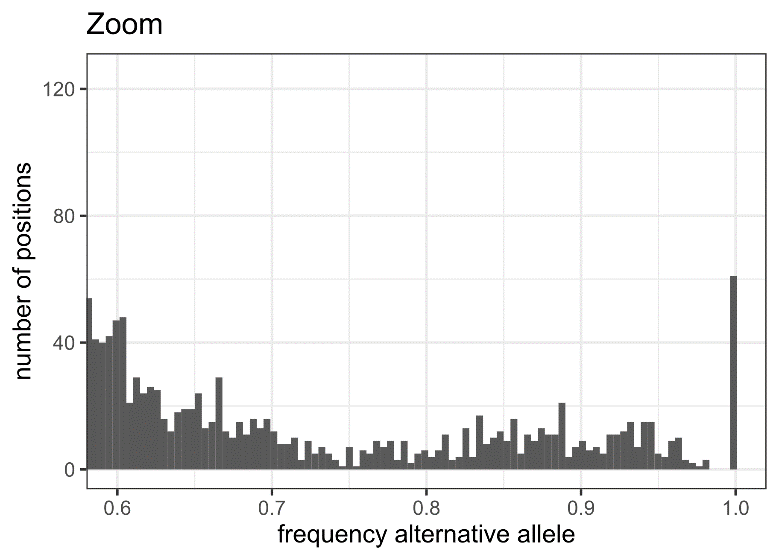


**C**

**B**


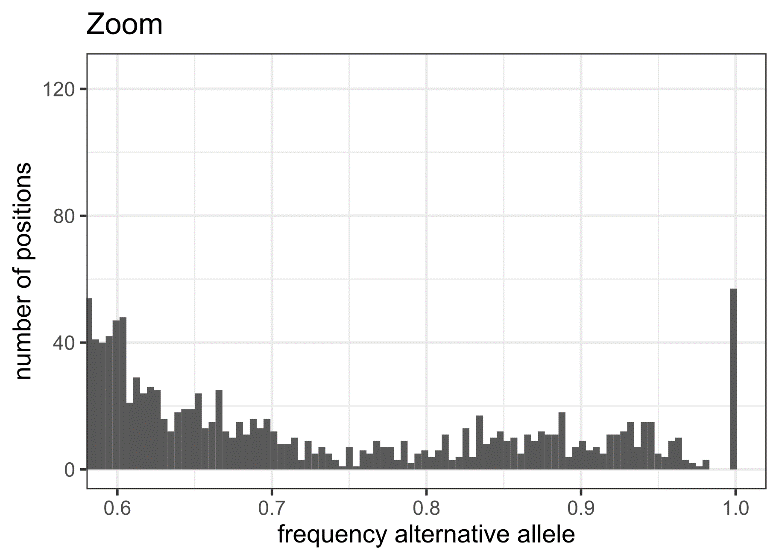

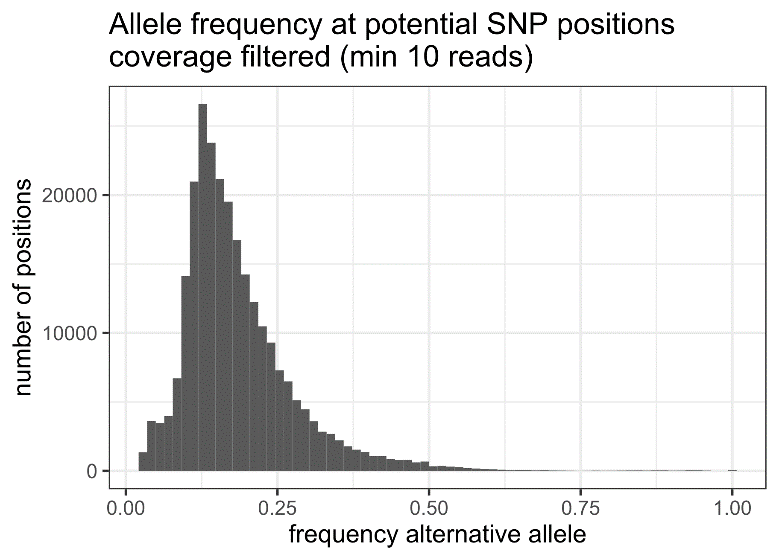


**E**

**D**


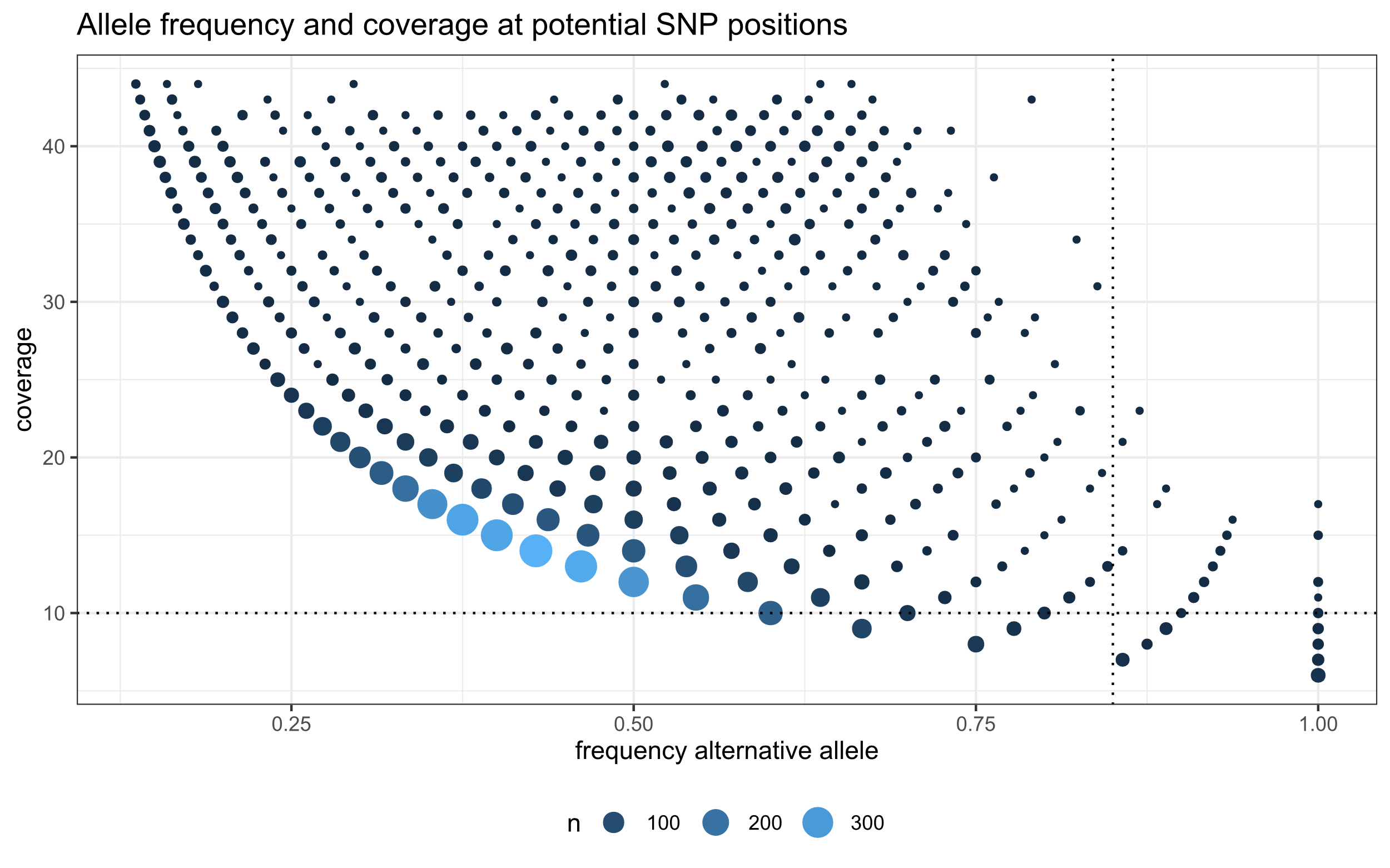


**Flongle HZ x13**

**A**


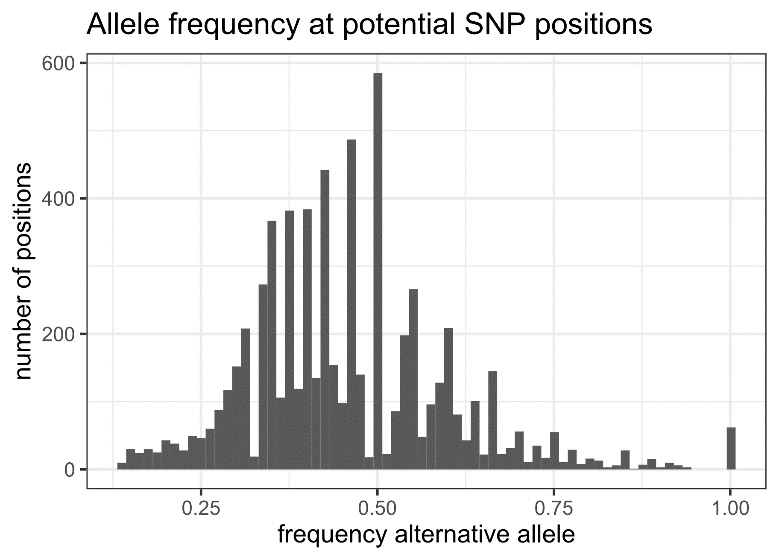


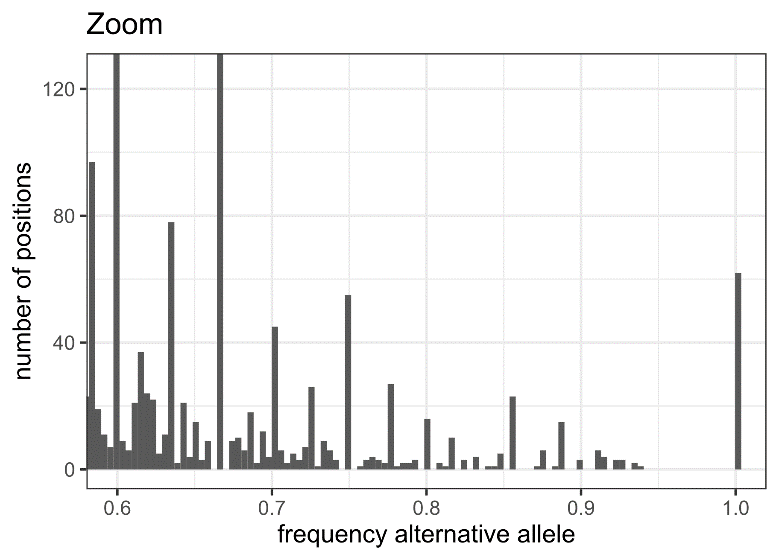


**C**

**B**


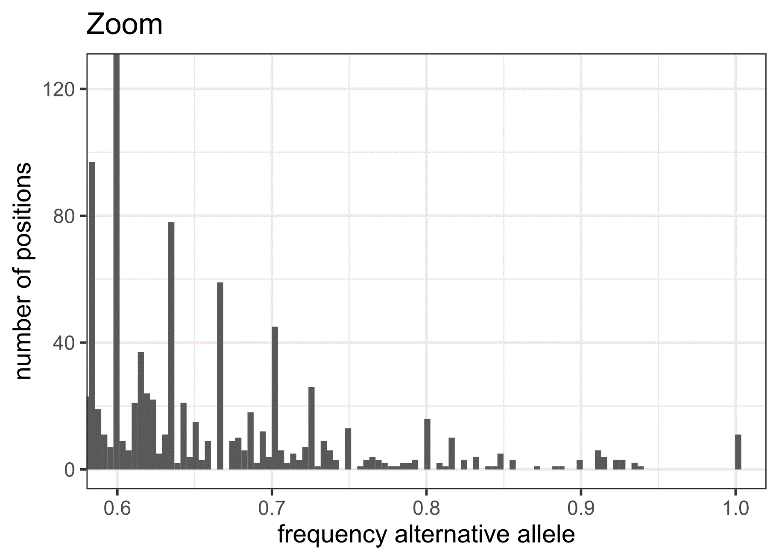

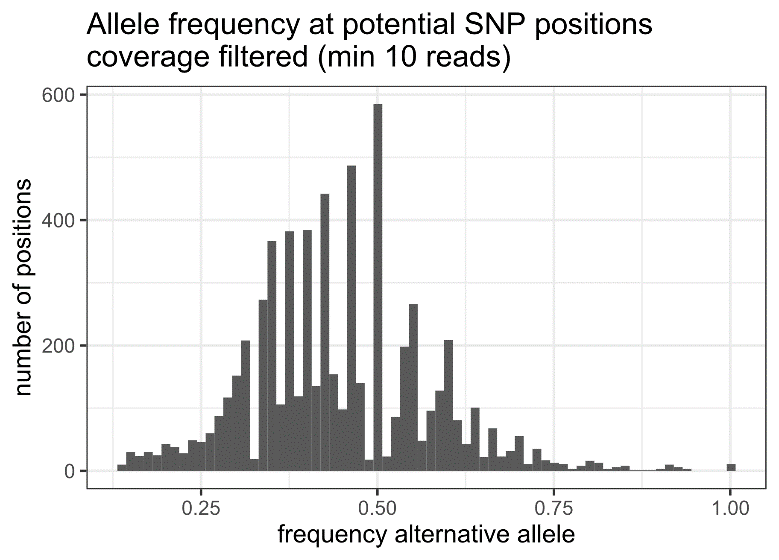


**E**

**D**
