# Supplementary material for: Towards Real-Time and Affordable Strain-Level Metagenomics-Based Foodborne Outbreak Investigations Using Oxford Nanopore Sequencing Technologies
Source: Front Microbiol. 2021 Nov 5;12:738284. doi: 10.3389/fmicb.2021.738284 (PMC8602914; doi:10.3389/fmicb.2021.738284)
Supplement: Supplementary Material 2 — Workflow of command lines for bioinformatics analyses used in this study. [file Table_2.DOCX]

Supplementary materials 2: Workflow of command lines for bioinformatics analyses used in this study

1. Example command line to run Metamaps

#!/bin/bash

#running Metamaps

metamaps mapDirectly -t 5 --all -r myDB/DB.fa -q run.fastq -o classification_results --maxmemory 10

metamaps classify -t 5 --mappings classification_results --DB myDB

#extracting reads

##obtaining list of taxons populated by reads

cat classification_results.EM.reads2Taxon | cut -f 2 | sort -u > unique

##extracting names of the reads assigned to each taxon

while read i; do grep $i'$' classification_results.EM.reads2Taxon | cut -f 1 > $i ; done < unique

##extracting reads using filterbyname.sh function from bbmap

filterbyname.sh in=run.fastq out=<taxon>.fastq include=t names=list_reads

2. Example command line to perform in silico DNA walking

#Running blast

#!/bin/bash

blastn -db genes.fasta –query reads.fasta -show_gis -task megablast -out reads_blastn_genes.txt -outfmt '6 qseqid sseqid pident length mismatch gapopen qstart qend sstart send bitscore stitle staxids'

blastn –db nucleotideNCBI –query reads.fasta –max_target_seqs 1 -show_gis -task megablast -num_threads 10 -out reads_blastn_nt.txt -outfmt '6 qseqid sseqid pident length mismatch gapopen qstart qend sstart send bitscore stitle staxids'

#Processing output (python 3)

#!/usr/bin/env python

import pandas as pd

import glob

##import genes = result from blastn on genes database.

genes=glob.glob('reads_blastn_genes.txt')

##import nt = result from blastn on nt database

nt=glob.glob('reads_blastn_nt.txt')

##create dataframe for genes

datagenes=pd.read_csv(genes[0],sep='/t', header=None, names=list(‘abcdefghijklmn’)

datagenes=datagenes.drop(columns=[ 'd','e','f','g','h','i','j','k','l','m','n','o'])

##modify the column of genes to have only the gene name -> split after double dots and keep only first part in this case

tempgenes=datagenes['b'].str.split(':', expand=True)

datagenes['gene']=tempgenes[0]

datagenes = datagenes.drop(columns=['b','c'])

datagenes = datagenes.drop_duplicates(subset=['a', 'gene'])

##create dataframe for nt

datant=pd.read_csv(nt[0],sep='\t', header=None,names=list('abcdefghijkl'))

dfnt = pd.DataFrame(datant)

##index the reads with genes hit

genesreads=datagenes['a']

idx_genesreads = pd.MultiIndex.from_arrays([genesreads],names=['reads'])

list_genesreads= idx_genesreads.unique(level=0)

##Look at the hits in nt for the reads that have a genes hit (only to genus level -> column k split after space and only first word kept)

minidfnt=dfnt.loc[dfnt['a'].isin(list_vfreads)]

minidfnt=minidfnt.drop(columns=['b','c','d','e','f','g','h','i','j','l'])

genus=minidfnt['k'].str.split(' ', expand=True)

minidfnt['k']=genus[0]

##Merge minidfnt and datagenes to get a table with read, organism hit from nt, gene hit from genes

finaldf= datagenes.merge(minidfnt,how='outer')

finaldf=finaldf.drop_duplicates(subset=['a', 'gene'])

finaldf=finaldf.drop(columns='a')

##Get count of number of occurence of gene / genus

size=finaldf.groupby(['gene', 'k']).size().reset_index()

3. Example command line to map Minion reads using bwa, followed by snp calling using samtools and generation of consensus sequence with masked low-quality regions using bcftools:

#!/bin/bash

#please pay attention to the software versions used in the manuscript, behaviour of samtools and bcftools is different for different versions

#Variables:

reference=<reference genome>

input_files=<directory with fastq.gz files>

mindepth=10

minallelefreq=0.85

alternativeallelethreshold=5

minmappingqual=50

#Read mapping

##indexing reference genome

bwa index $1

##mapping reads

for i in $input_files/*fastq.gz

do

bwa mem -x ont2d -t $N $reference $i > $(basename $i .fastq.gz).sam

done

##sorting sam files, outputting bam files

for i in *sam

do

samtools sort -@ 20 -o ${i/.sam/.bam} $i

done

##indexing bam files

for i in *bam;

do

samtools index $i

done

##cleaning up

mkdir bam 2>/dev/null

mv *sam *bam *bai bam 2>/dev/null

#SNP calling and generation of vcf/bed files to find low-quality positions

##generation of mpileup files containing info on all positions with a non-zero coverage

for i in bam/*bam

do

bcftools mpileup -a FORMAT/AD,FORMAT/DP -A -B --ff SECONDARY -q 0 -Q 0 --skip-indels -f $reference $i > ${i/.bam/}.mpileup

done

##generation of vcf's with the potential SNP positions

##this file is used to call high-quality SNPs and to find and mask positions that fail alternative allele filtering criteria

for i in bam/*mpileup

do

bcftools filter -i "FORMAT/AD[0:1] > $alternativeallelethreshold" $i -O v > $(basename $i .mpileup)_snps.vcf

#AD[0:1] means second allele of the first sample

done

##generation of vcf's with all positions

##this file is used to find and mask positions with inferiour mapping quality and coverage

##zero-coverage positions are not included in this file

for i in bam/*mpileup

do

cat $i | bcftools call -V indels -m --ploidy 1 - > $(basename $i .mpileup)_allsites.vcf

done

##Creating bed files and zipping vcf files

for i in *_allsites.vcf

do

cat $i | awk '{OFS="\t"; if (!/^#/){print $1,$2-1,$2,$4"/"$5,"+"}}' > ${i/.vcf/}.bed

done

for i in *.vcf

do

bgzip $i

done

##Filtering the high-quality SNP's

##please note that positions in the regions failing the mapping quality filter are still included in the PASS vcf file

##such positions are masked when creating consensus sequence

for i in *snps.vcf.gz

do

bcftools filter $i -e "FORMAT/DP<$mindepth || FORMAT/AD[0:1]/FORMAT/DP<$minallelefreq" > ${i/.vcf.gz/}_PASS.vcf

done

##putting generated files in a separate directory

mkdir vcf

mv *_PASS.vcf vcf

#Generating bed files with the positions that should be masked based on the depth, allele frequency and mapping quality criteria

##allele frequency

for i in *snps.vcf.gz

do

bcftools filter $i -i "FORMAT/AD[0:1]/FORMAT/DP < $minallelefreq" | awk '{OFS="\t"; if (!/^#/){print $1,$2-1,$2,$4"/"$5,"+"}}' > ${i/.vcf.gz/}_tomask_frequency.bed

done

##mapping quality

for i in *allsites.vcf.gz

do

bcftools filter $i -i "INFO/MQ<$minmappingqual" | awk '{OFS="\t"; if (!/^#/){print $1,$2-1,$2,$4"/"$5,"+"}}' > ${i/.vcf.gz/}_tomask_mq.bed

done

##low coverage (zero coverage positions not included)

for i in *allsites.vcf.gz

do

bcftools view $i -i "FORMAT/DP<$mindepth" | awk '{OFS="\t"; if (!/^#/){print $1,$2-1,$2,$4"/"$5,"+"}}' > ${i/.vcf.gz/}_tomask_depth.bed

done

##zero coverage

###generating a genomefile

cat $reference | awk '$0 ~ ">" {print c; c=0;printf substr($0,2,100) "\t"; } $0 !~ ">" {c+=length($0);} END { print c; }' > $reference.genomefile

###calling positions with a coverage of 0 (are not part of the allsites.vcf files)

for i in *allsites.bed

do

bedtools complement -g $reference.genomefile -i $i | grep -v "0$(printf '\t')0" > ${i/.bed/}_tomask_zerodepth.bed

done

##Putting away the vcf files

mkdir intermediate_vcf 2>/dev/null

mv *vcf.gz intermediate_vcf/

##Fusing the bed files per isolate/sample to create "tomask.bed" file used to mask low-quality positions

for i in *_tomask_frequency.bed

do

name=`basename $i _snps_tomask_frequency.bed`

echo $i

multiIntersectBed -i $i ${name}_allsites_tomask_depth.bed ${name}_allsites_tomask_zerodepth.bed ${name}_allsites_tomask_mq.bed > ${name}_tomask.bed

done

##Putting away the bed files

mkdir intermediate_bed 2>/dev/null

mv *_snps_tomask_frequency.bed *_allsites.bed *_allsites_tomask_depth.bed *_allsites_tomask_mq.bed *_allsites_tomask_zerodepth.bed intermediate_bed

##Putting the final bed files in a separate directory

mkdir masking_bed 2>/dev/null

mv *tomask.bed masking_bed

#Creating consensus fastas and masking low-quality regions

##Indexing fasta file with picard tools

if [ ! -s ${reference/.fasta/.dict} ]

then

java -jar picard.jar CreateSequenceDictionary -REFERENCE $reference -OUTPUT ${reference/.fasta/.dict}

fi

##Creating consensus fastas and masking

mkdir consensusandmaskedfastas

for vcf in vcf/*vcf

do

bgzip $vcf

tabix $vcf.gz

done

for vcf in vcf/*vcf.gz

do

name=`basename $vcf _snps_PASS.vcf.gz`

echo $name

bcftools consensus -f $reference -m masking_bed/${name}_tomask.bed $vcf > consensusandmaskedfastas/${name}_masked.fasta

done
